# Pyrrole-Mediated Peptide Cyclization Identified through Genetically Reprogrammed Peptide Synthesis

**DOI:** 10.3390/biomedicines6040099

**Published:** 2018-10-30

**Authors:** Klaas W. Decoene, Willem Vannecke, Toby Passioura, Hiroaki Suga, Annemieke Madder

**Affiliations:** 1 Department of Organic and Macromolecular Chemistry, Ghent University, Krijgslaan 281 S4, 9000 Ghent, Belgium; Klaas.decoene@ugent.be (K.W.D.); willemvannecke@gmail.com (W.V.); 2 Department of Chemistry, Graduate School of Science, The University of Tokyo, 7-3-1 Bunkyo-ku, Tokyo 113-0033, Japan; toby@chem.s.u-tokyo.ac.jp (T.P.); hsuga@chem.s.u-tokyo.ac.jp (H.S.)

**Keywords:** constrained peptides, flexible in vitro translation, furan oxidation, peptide cyclisation

## Abstract

Flexible in vitro translation (FIT) was used as a screening method to uncover a new methodology for peptide constraining based on the attack of a nucleophilic side-chain functionality onto an oxidized furylalanine side chain. A set of template peptides, each containing furylalanine as furan-modified amino acid and a nucleophilic residue (Cys, His, Lys, Arg, Ser, or Tyr), was produced through FIT. The translation mixtures were treated with *N*-bromosuccinimide (NBS) to achieve selective furan oxidation and subsequent MALDI analysis demonstrated Lys and Ser as promising residues for cyclisation. Solid-phase peptide synthesis (SPPS) was used to synthesize suitable amounts of material for further in-depth analysis and characterisation. It was found that in the case of the peptide containing lysine next to a furylalanine residue, a one-pot oxidation and reduction reaction leads to the generation of a cyclic peptide featuring a pyrrole moiety as cyclisation motif, resulting from the attack of the lysine side chain onto the oxidized furylalanine side chain. Structural evidence was provided via NMR and the generality of the methodology was explored. We hereby expand the scope of our previously developed furan-based peptide labeling and crosslinking strategy.

## 1. Introduction

From a pharmaceutical perspective, peptides occupy a transition zone between small molecule chemicals and larger biologics [1]. Compounds in this intermediate size range can theoretically combine the advantages of biologics (e.g., the specificity of monoclonal antibodies and their ability to disrupt protein-protein interactions) with the desirable aspects of small molecule drugs (e.g., oral availability and biostability). In particular, relatively small peptides (up to ~15 amino acids) constrained through cyclizing linkages are capable of interfering with protein–protein interactions, exhibit high target specificity, and can be highly resistant to serum proteases, and yet are small enough to be orally available and avoid immune responses [1,2]. For these reasons, several constrained peptides are used as therapeutics [1] (e.g., octreotide, cyclosporine A, nisin) and more are in late-stage clinical trials [2]. Moreover, new approaches for constraining the inherently flexible conformation of short peptides are highly sought after and form a subject of continuous investigation.

From an organic synthetic perspective, constraining peptide structure can be achieved in numerous ways. Four conceptually different approaches can be adopted to obtain cyclic peptides [1], depending on the functional groups present in the side chains of the envisaged sequence: head-to-tail, head-to-side-chain, side-chain-to-tail, and side-chain-to-side-chain connection. In this way, not only head-to-tail cyclic peptide construction but also loop stabilization and stabilization of alpha helices through so-called peptide stapling can be achieved by creating covalent connections between two of the side chains of a specific peptide sequence. Methods can also be conceptually subdivided into either two component systems, in which a bireactive external component is added to react with two functional groups in the peptide (commonly side-chain moieties), or one-component systems, in which two moieties in the peptide react to form a cyclizing link. When considering the specific nature of the side-chain functionalities of the relevant amino acids, further distinctions can be drawn based on whether two natural amino acid residues, one natural and one unnatural amino acid residue, or two unnatural amino acids are involved in the reaction. 

A number of chemistries used in two-component systems are based on the use of two unnatural amino acids that react with double “click” linkers [3]. Alternatively, reactions using two canonical amino acids include double thioether [4] linkages via reaction with Cys residues. Arylation [5] and alkylation [6,7] chemistry on both Lys and Cys side chains have also been reported. In view of the higher complexity of such two-component systems and the possibility for formation of regio-isomeric products when applying unsymmetrical linker components, alternative, one-component systems have been developed. 

One-component approaches where two natural amino acids are involved in the peptide cyclisation include lactam formation between Lys and Glu/Asp [8] and formation of a disulphide bridge between two Cys residues [9] in addition to enzymatic cyclisation strategies, for example, between Lys and Trp [10]. However, one-component systems employing two natural amino acids inherently lack selectivity due to the possible presence of other natural residues in the sequence of interest. Therefore, chemistries using two unnatural amino acids have been developed, including ring-closing metathesis (RCM) hydrocarbon linkages [11], copper-catalysed azide alkyne click (CuAAC) [12] reactions, alkene-tetrazole [13] cycloaddition, glaser coupling [14], and oxime formation [15]. It is, however, not always straightforward to incorporate two unnatural amino acids in a peptide sequence. Therefore, strategies using one unnatural and one naturally occurring amino acid functionality (thereby combining a degree of selectivity with ease of synthesis) have gained increasing attention, such as the formation of a thioether [16] between a specifically located Cys residue and a second residue featuring an alkylating moiety, or peptide macrocyclization via reaction of a noncanonical aldehyde moiety and the peptide N terminus [17]. 

In addition to the chemical peptide synthesis approaches discussed above, biotechnological techniques for the generation of constrained peptides have recently been developed. In particular, genetic code reprogramming approaches, such as flexible in vitro translation [18] (FIT), allow for the incorporation of diverse unnatural amino acids into peptides synthesized ribosomally, thereby enabling diverse one-component cyclizing chemistries in translated peptides [19,20,21,22,23,24]. In a FIT reaction, one or more unnatural amino acids are aminoacylated onto specific tRNAs, which are then introduced into a fully reconstituted in vitro translation reaction deficient for one or more natural amino acids. In this way, specific unnatural amino acids of interest can be translated in place of their natural analogues. The aminoacylation reaction itself is achieved through the use of flexizymes (flexible tRNA acylation ribozymes), which catalyze the aminoacylation of tRNA 3′-terminal hydroxyls using an amino acid substrate activated via an ester (or thioester) leaving group. This highly versatile approach has been used for the synthesis of peptides containing diverse unnatural amino acids including multiple cyclizing chemistries [19,20,21,22,23,24].

**Figure 1:**
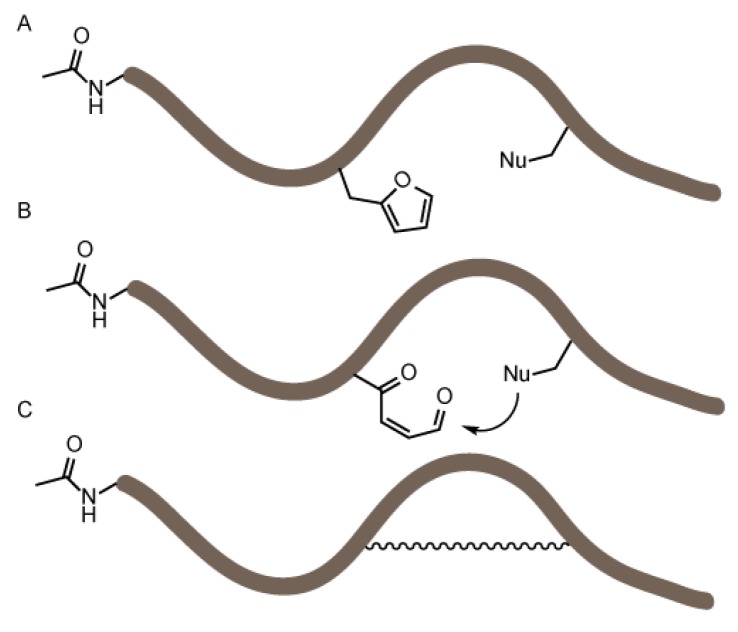
Schematic overview of the objective of the flexible in vitro translation screening experiment. A set of peptides was produced by flexible in vitro translation (FIT) (**A**) all bearing a furan amino acid and a nucleophilic residue. In a second stage, the furan moiety was oxidized (**B**) and nucleophiles can potentially react with the keto-enal moiety. The peptides were analyzed via MALDI-TOF mass spectrometry to identify any formed constrained peptides (**C**).

In the context of our previous work on furan-based peptide labeling [25,26] and peptide/protein crosslinking [27], we became interested in exploiting FIT for the exploration of the potential utility of furan side chains in a peptide-constraining context. To identify which of the canonical nucleophilic amino acids is prone to react with an oxidized furan moiety, we made use of FIT to produce a set of peptides encompassing both a furan amino acid and a nucleophilic amino acid that could potentially react to form a side-chain-to-side-chain linked structure (Figure 1).

In the current work, we show that a furan amino acid can be incorporated via FIT and, following peptide translation, selective furan oxidation within the translated peptides can be achieved using *N*-bromosuccinimide (NBS). It was shown that upon NBS treatment, a Lys residue, occurring in the peptide sequence, induces formation of a cyclic imine at lower pH conditions. Upon reduction of the resulting conjugate, a pyrrole-containing cyclic peptide was isolated. We here provide structural evidence (NMR) for the formation of the pyrrole unit as a cyclisation motif and explore the generality of this new peptide-constraining methodology.

## 2. Experimental Section

### 2.1. Synthesis of Cyanomethyl Ester (CME)-Activated Amino Acid Substrates

The synthesis of *N*-acetyl phenylalanine was reported previously (see Supplementary Materials Sections 2 and 12) [28]. For synthesis of CME-activated 2-furanyl alanine (see Supplementary Materials Section 1 for details), Boc-l-2-furylalanine. DCHA (1 part) was suspended in 5–10 volumes of cold t-butyl methyl ether. 10% of phosphoric acid was added under stirring until the dicyclohexylammonium (DCHA) salt was completely dissolved and two clear phases appeared. The pH of the lower aqueous phase was around 2–3. The organic phase was isolated and washed once with 2 volume parts of phosphoric acid 10% and 3 times with 2 volume parts of water. The pH of the aqueous phase was ≥4. TLC (2:1 hexane/EtOAc) was used to check whether the amino acid was liberated from its DCHA salt. The organic phase was dried over anhydrous sodium sulfate, filtered off, and evaporated to dryness in vacuo to obtain the free amino acid. Boc-l-2-furylalanine was dissolved in 300 μL of chloroacetonitrile, 1,2 eq of DIPEA was added, and the mixture was reacted overnight at room temperature. Completion of the reaction was followed by TLC (2:1 hexane/EtOAc). The reaction mixture was transferred to a separation funnel of 100 mL and extracted with 50 mL of Et_2_O. The organic phase was washed with 3 × 50 mL of 1M HCl (aq), 2 × 50 mL with saturated NaHCO_3_ (aq), 2 × 10 mL with saturated NaCl (aq). The organic phase was dried over Na_2_SO_4_ and evaporated to dryness. Flash chromatography was used for purification of the compound (2:1 hexane/EtOAc). The collected fractions were evaporated to dryness. The purified compound was then dissolved in 4 M HCl/dioxane and stirred for 10 min on ice, followed by 30 min at room temperature. The reaction was followed by TLC (2:1 hexane/EtOAc). The reaction mixture was evaporated to almost complete dryness. Cold Et_2_O was added to precipitate the amino acid. The Boc-group was removed by dissolving the peptide in 4 M HCl/dioxane and keeping the solution on ice for 30 min. After reaction, the HCl/dioxane is partially removed by evaporation, with subsequent addition of cold diethylether to precipitate the amino acid. After centrifugation, the supernatant was discarded and the amino acid was obtained after drying in vacuo.

### 2.2. Flexible In Vitro Translation

Flexizymes and tRNAs were synthesized as previously described [18]. Aminoacylation of the elongator tRNA with furylalanine, 1 µL 500 mM HEPES-KOH buffer of pH 7.5, and 250 µM flexizyme (eFx) and 250 µM tRNA were added to 3 µL of milliQ H_2_O. The mixture (6 µL total volume) was placed in a heat block at 95 °C for 2 min. Afterwards, 2 µL 3M MgCl was added and the tube was mixed well and placed at room temperature for 5 min. The tube was placed on ice for several minutes and subsequently 2 µL of 25 mM furylalanine-CME (DMSO solution) was added. The solution was mixed and incubated on ice for 6 h. For the aminoacylation of initiator tRNA with *N*-acetylated phenylalanine, the same protocol was used except for the final incubation time on ice, which was 2 h. After EtOH precipitation of aminoacyl-tRNA, the mixture was divided in two (5 µL each). Then, 20 µL of 0.3 M NaOAc and 50 µL of EtOH were added and the solution was mixed well. The solution was centrifuged at 13 krpm, 25 °C for 15 min, and the supernatant was discarded. Next, 30 µL of 70% EtOH containing 0.1 M NaOAC (pH 5.2) was added to the pellet and vortexed for 10 s, centrifuged for 5 min, and the supernatant was discarded. This last step was repeated and finally 20 µL of 70% EtOH was added and the sample was centrifuged for 3 min. The supernatant was discarded, the lid was opened, and the tube was covered with a clean paper towel and the sample was left to dry for 5–10 min.

Genetically reprogrammed translation was performed in a FIT reaction at a final scale of 2.5 µL at 37 °C for 30 min as previously described [18]. After translation, MALDI-TOF MS analysis was performed as previously described [18] using an UltraFlex instrument (Bruker, Billerica, MA, USA).

### 2.3. Solid-Phase Peptide Synthesis

Peptides were synthesized using standard Fmoc chemistry on chlorotrityl resin (see Supplementary Materials Sections 6 and 12). The automated peptide synthesis was performed on either a MultiPep (Intavis, Tübingen, Germany) or Syro (Multisyntech, Witten, Germany) device. Synthesis with double couplings was performed with HBTU as coupling reagent, 5 equivalents amino acid and DIPEA in NMP as base. Fmoc deprotection was carried out with piperidine in DMF. Following the automated peptide synthesis, the *N*-terminus was acetylated with a mixture of 5% acetic acid anhydride and 6% 2,6-lutidine in DMF in a 30 min reaction at room temperature. A mixture of 95% trifluoro acetic acid (TFA), 2.5% triisopropyl silane, and 2.5% H_2_O was used to cleave the peptide from the resin. Peptides were subsequently precipitated in cold methyl tert-butyl ether. Purification of the synthesized peptides was done on an Agilent 1100 Series HPLC instrument equipped with a Phenomenex Luna C18(2) at 35 °C flow rate of 4.5 mL/min. The column was eluted with a gradient from 100% H_2_O with 0.1% TFA to 100% acetonitrile in 20 min. 

## 3. Results and Discussion

### 3.1. FIT-Based Synthesis of Furan-Modified Peptides

Taking advantage of the utility of FIT for the facile synthesis of diverse peptides at small scale, a screening experiment was designed to identify amino acids able to react with an oxidized (activated) furan amino acid. Commercially available furylalanine (Fua), which is isosteric with His and isoelectronic with respect to Tyr, was selected as the furan-containing amino acid. For this screening experiment, simple template peptides were designed containing no other reactive functional groups aside from those functionalities to be examined for their capacity to intramolecularly react with the (oxidized) furan moiety. Since the *N*-terminus is also a potential nucleophile and we were interested in reactions of the oxidized furan moiety and the side chains of natural amino acids (peptide-constraining) rather than head-to-side-chain macrocyclization, each peptide was translated with an acetylated *N*-terminus through the use of *N*-acetyl Phe (AcF). To favour eventual cyclisation events by enhancing the proximity between the two reactive groups, a Pro residue was positioned in between. The final template peptide sequence used was AcPheAlaGlyAlaFuaGlyProGly**X**AlaGlyAla with **X** being a natural residue featuring a nucleophilic side chain Cys, His, Lys, Arg, Ser, or Tyr.

**Figure 2:**
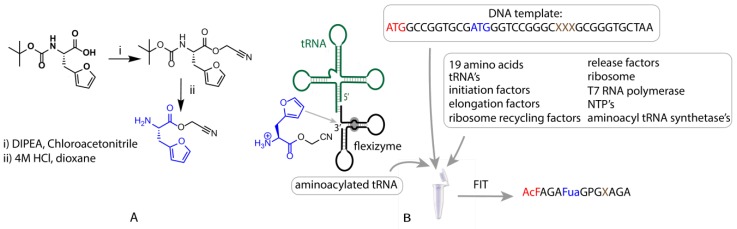
Schematic representation of the flexible in vitro translation system used for expression of furylalanine-containing peptides. On the left side (**A**), the structure and synthesis of the CME-activated furylalanine is presented along with a simplified structure of the flexizyme eFx and a tRNA molecule (green) (**B**). The translated peptides contained an *N*-acetylated Phe (red) at the *N*-terminus and a Fua (blue), as well as a range of nucleophilic residues (brown). For all amino acid residues, one-letter codes are used in this table except for furylalanine, abbreviated as Fua.

FIT-mediated translation of peptides initiated with *N*-acetyl Phe has been described previously [28], however, to the best of our knowledge, use of this approach for translation of Fua has not been reported. To achieve this, cyanomethylester (CME)-activated Fua was synthesized starting from boc-furylalanine, through esterification with chloroacetonitrile, followed by boc deprotection (Figure 2A, Supplementary Materials Section 1). This activated Fua was then charged onto a tRNA for decoding of the Met AUG elongator (i.e., not initiator) codon in a flexizyme (in this case eFx)-catalysed reaction (Figure 2B). A similar process was used for *N*-acetyl Phe with the exception that a tRNA specific for peptide initiation was used. Due to the fact that the acetyl-Phe residue is located at the *N*-terminus, both unnatural residues are incorporated in response to the same codon (ATG), a consequence of the difference between translation initiation and elongation. Addition of these two unnatural amino-acid-charged tRNAs to an in vitro translation reaction deficient for Met thus produced a genetic code in which *N*-acetyl Phe replaced the initiating Met, with Fua replacing any downstream Met residues and all other codons encoding their cognate natural amino acid (Figure 2B, Supplementary Materials Section 3).

To confirm translation of the test templates under the genetic code used (both described above), small-scale translation reactions (2.5 µL each) were assessed by MALDI-TOF MS. Table 1 lists the calculated expected mass for each peptide, followed by the detected ions (original MALDI-TOF mass spectra are included in the Supplementary Materials, Figures S4.1–S4.6). These experiments demonstrate successful translation of all peptides, with the M + K^+^ and M + Na^+^ peaks detected in all cases, and the M + H^+^ peaks also found for the more easily protonated species (i.e., those including His, Lys, and Arg). The use of the FIT system allowed us to have rapid access to the peptide library we envisaged for this study on a small scale. Once the CME-activated amino acids are synthesized, the tRNA aminoacylation, peptide translation, oxidation, purification, and MALDI-TOF analysis can all be done in a single day.

### 3.2. NBS Oxidation of Furan-Containing Peptides Obtained through FIT

One of the advantages of the furan methodology relies in the fact that furan as such is not reactive towards nucleophiles present in a biological system, but reactivity can be unveiled by oxidation. Therefore, the translated, furan-containing peptides undergo an oxidation step prior to screening for cyclisation reactions. In this work, *N*-bromosuccinimide (NBS) was used to selectively oxidize the furan moiety in the template peptides [25], with oxidation and/or cyclisation being monitored by MALDI-TOF. Since the furan moiety as well as the nucleophilic residue are present in a single peptide, we carefully analysed the spectra for the presence of products resulting from potential intramolecular reactions. In Table 2, the results of the MALDI-TOF analysis are shown (original mass spectra are added in the Supplementary Materials Section 5). 

For the Cys-containing template peptide **1C**, the original protonated mass was not observed (nor were sodium or potassium adducts), but a new cysteinylation product (1214.97 Da) was detected in addition to its furan oxidation product +16 Da (1230.97 Da). While the Cys thiol moiety is the most nucleophilic functional group tested, these results suggest that under the oxidizing conditions used for formation of the keto-enal group, disulphide bond formation between the peptide Cys and free Cys in solution prevents reaction with the oxidized furan. In the case of the His-containing template peptide **1H,** next to the potassium adduct of **1H**, also the furan oxidation product **2H** was identified, but no product resulting from a potential cyclization was observed. The mass spectrum for the Arg-containing template peptide **1R** showed the M + H^+^ peak as well as the potassium adduct (M + K^+^), along with a product resulting from oxidation **2R**. For the Tyr-containing peptide **1Y**, a mass corresponding to dibromination of Tyr in the starting peptide as well as the furan oxidation product thereof were observed, not unexpected due to the use of a huge excess of NBS. For the Lys- as well as Ser-containing template peptides **1K** and **1S**, we observed the sodium and potassium adducts of the starting peptides as well as the potassium adduct of the furan oxidation product. Additionally, in both cases, a particular signal corresponding to M + H − 20 Da was detected (highlighted in grey in Table 2). This observation of a reduced mass could indicate, for example, an oxidation (resulting in a peptide mass M + 16), followed by an intramolecular cyclization reaction accompanied by the loss of water (resulting in a peptide mass M + 16 − 18), and an additional loss of water during ionisation (M + 16 − 18 − 18 = M − 20).

### 3.3. Peptide Scale up by SPPS and Subsequent NBS Oxidation

**Figure 3:**
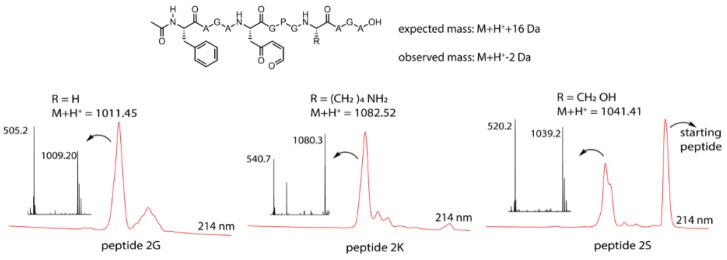
Top: general structure of the oxidized peptides **2**, the expected mass (in case of oxidation only) and the observed mass. Bottom: M + H^+^ mass for the individual peptides, LC chromatogram at 214 nm (zoom of the relevant region) red and the MS spectrum of the oxidation peak for the three oxidized peptides.

Based on the results of the FIT screening, the Lys- and Ser-containing template peptides **1K** and **1S** were resynthesized via solid-phase peptide synthesis (SPPS) in order to generate sufficient material for more detailed characterisation of the reaction following oxidation (see Supplementary Materials Section 6). In addition, a control peptide with a Gly residue rather than a nucleophilic residue was synthesized. Similar to the FIT screening, the peptides were treated with NBS to oxidize the furan moiety. To render the oxidized furan moiety more electrophilic and to promote imine formation (in the case of peptide **2K**), this reaction was performed in the presence of NaOAc buffer of pH 5.2 as per Malins et al. [17]. In Figure 3, the structure of the three resulting peptides is shown (top) with the LC chromatogram (red) as well as the MS spectrum corresponding to the oxidation peak (bottom) for each of the peptides.

From the MS spectrum corresponding to the LC signal of the product formed upon oxidation, each time a mass of −2 Da compared to the M + H^+^ mass can be observed for the three peptides. This is 18 Da lower than the expected +16 Da mass for the oxidation product (Figure 3), which can be explained by oxidation of the furan moiety followed by loss of water (+16 Da − 18 Da = −2 Da). The obtained mass data should, however, be interpreted with care. Indeed, the observed loss of water can be the result of an intramolecular reaction, thus indicating formation of a new species resulting from a cyclisation event, but can also occur during the ionisation (the signal thus corresponding to the oxidized product without further cyclisation). To determine whether the M + H^+^ − 2 Da peak and the M + H^+^ + 16 Da peak originated from one and the same compound in the LC, the chromatograms for both extracted ions (−2 Da and +16 Da) were compared. The results indicated that for peptides **2G** and **2S**, both ions originated from the same (oxidized but not cyclized) compound in the LC, indicating that the M + H^+^ − 2 Da peaks resulted from the ionisation process and were artefacts of mass spectrometry. However, for peptide **2K**, it was clear that the M + H^+^ − 2 Da and M + H^+^ + 16 Da ions originated from different products, implying that the M + H^+^ − 2 Da peak may be due to imine formation (see Supplementary Materials Section 7 for complete chromatograms and extracted ion chromatograms). It should be noted that in the chromatogram of the Ser-containing peptide template, a substantial amount of native (nonoxidized) peptide was observed, resulting from incomplete oxidation due to a greater amount of starting material in this case.

### 3.4. Identification of a Pyrrole Moiety as Cyclisation Motif

**Figure 4:**
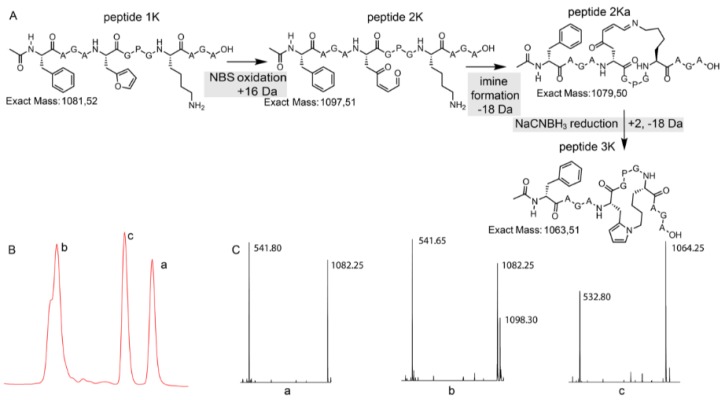
(**A**) Schematic overview with structural representation of the one-pot peptide oxidation and reduction reaction. (**B**) A zoom-in of the LC chromatogram (214 nm) of the reaction mixture is shown. (**C**) A zoom-in of the ESI-MS spectra (positive mode) of the three prominent peaks. a: starting peptide, b: oxidation products, c: pyrrole-constrained peptide.

To quench the rather labile imine-constrained peptide (Figure 4, peptide **2Ka**), we decided to try and reduce it to the corresponding secondary amine with sodium cyanoborohydride. This reduction would theoretically result in a product with the same mass as the original peptide. However, MS analysis of the sample, in which NBS oxidation was followed by NaCNBH_3_ reduction, revealed the presence of a compound with a mass corresponding to M + H^+^ − 18 Da (Figure 4). This mass can be explained by an additional subsequent attack of the formed amine (upon reduction) on the remaining keto functionality followed by aromatization through loss of water (see Supplementary Materials Section 8). 

In the chromatogram shown in Figure 4, three major peaks can be observed. The peak at the far right (a) corresponds to the starting peptide which was not oxidized. Signal b in part B of Figure 4 can be correlated to oxidation products. The MS spectrum of peak b indicates two products, the expected oxidized peptide with a mass of 1098.30 Da and a product with the mass of the starting peptide. This last product can be explained by the fact that oxidation took place and an imine was formed (with loss of water) which was further reduced to a secondary amine upon NaBH_3_CN treatment without further reaction to the pyrrole-constrained peptide. Finally, the MS spectrum of peak c shows a mass of 1064.25 Da, which can be correlated with the formation of a pyrrole-constrained peptide. We decided to further explore the scope of this pyrrole-based cyclisation while at the same time generating additional structural evidence for formation of the pyrrole moiety as cyclisation element.

### 3.5. Extending the Scope of Pyrrole-Mediated Cyclisation: Varying the Positioning and Conformation within the Template Peptide

**Figure 5:**
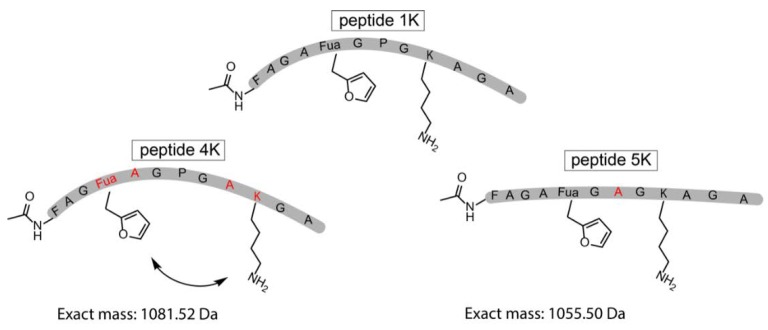
Schematic representation of the original template peptide (peptide **1K**) and 2 variations on the template peptide which were synthesized by SPPS. In peptide **4K**, the distance between the two reacting residues was increased, and in peptide **5K**, the Pro was exchanged for an Ala residue. The altered residues (Fua, A, K) in peptides **4K** and **5K** compared to peptide **1K** are depicted in red.

The original template peptide used in this work featured a Pro positioned in between the Fua and the Lys, resulting in a form of preorganisation of the peptide conformation inducing proximity between the reactive moieties. To demonstrate that the pyrrole-constrained peptide formation is not limited to the specific peptide sequence chosen here, two additional variants were synthesized via SPPS (Figure 5, Supplementary Materials Section 9). In one of these (peptide **4K**), the distance between the two reacting residues was increased by interchanging both Fua and Lys with a neighbouring Ala. On the other hand, in peptide **5K**, the internal Pro residue was replaced by an Ala.

**Figure 6:**
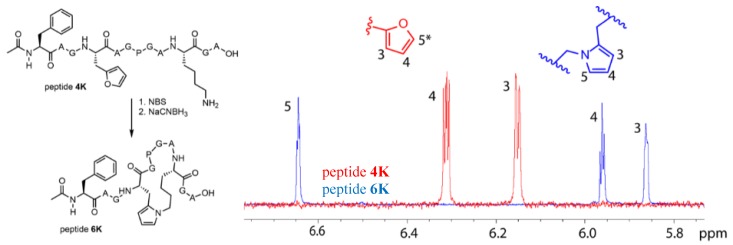
Structure of peptide **4K** and cyclisation product **6K** (**left**). Zoom of the ^1^H NMR spectrum of peptides **4K** (red) and **6K** (blue) between 5.8 and 6.8 ppm combined with the structures of the furan and pyrrole unit (**right**). The signals corresponding to the protons on C3, C4, and C5 are indicated except for the signal on C5* of the furan moiety which has a chemical shift larger than 7 ppm and is not shown in this figure.

The pyrrole-mediated, peptide-constraining reaction proved successful in the case of both peptide **4K** and **5K**, and LCMS data for the oxidation and reduction reactions towards the cyclic peptides **6K** and **7K** are included in the Supplementary Materials (Figures S10.1–S10.3). These one-pot oxidation and reduction reactions were also repeated at larger scale for peptide **4K** to allow structural characterisation of the cyclised product. The optimized reaction for the formation of **6K** was used to determine the conversion via HPLC peak integration (Figure S10.2). It was shown that near-complete conversion of the starting peptide to the oxidized products was achieved. However, the oxidized products did not convert completely to **6K**. The total conversion of **4K** to **6K** was 32%. The pyrrole compound (peptide **6K**) was purified by RP-HPLC and after lyophilization, the product was resolubilized in 90/10 H_2_O/D_2_O for NMR analysis on a 700 MHz (Bruker) spectrometer, confirming the structure of the pyrrole cyclized product. A zoom of the ^1^H NMR overlay spectrum of **4K** and **6K** is provided in Figure 6. The three expected signals for the pyrrole unit are present, a triplet for the proton on C4 and a hidden triplet for C3 and C5. For the furan unit in starting material **4K**, the signals of the protons on C3 and C4 are present; the signal for the proton on C5* is not visible in this zoom since its chemical shift is larger than 7 ppm. Complete NMR spectra (^1^H, TOCSY, ROESY, HSQC) with assignment of the signals for peptide **6K** and full ^1^H spectrum for **4K** are included in the Supplementary Materials (Section 11).

The *N*-termini of the peptides used in this study were masked to allow investigating the reactivity of natural nucleophilic amino acid side chains. As the *N*-terminal amine is more reactive than the ε-amino group of the lysine side chain, we expect that also pyrrole-constrained head-to-side-chain peptides can potentially be generated through this method.

## 4. Conclusions

In summary, a new pyrrole-mediated peptide cyclisation methodology was developed using FIT as an initial screening strategy. We here have shown the successful translation of template peptides containing both a furylalanine and a nucleophilic side chain residue using FIT. Oxidation of the furan moiety was achieved with NBS and MALDI-TOF analysis was used to screen for cyclized peptides. Structural analysis using NMR spectroscopy allowed confirming the formation of the pyrrole moiety as cyclisation motif. Further variation of the peptide structure indicates the generality of this novel methodology which thus promises to be useful in a broader context for the efficient cyclisation of peptides containing a furylalanine moiety and a suitably positioned lysine residue.

## Figures and Tables

**Table 1:** MALDI-TOF analysis of the translated peptide library. For all amino acid residues, one-letter codes are used in this table except for furylalanine, which is abbreviated as Fua. Color codes are in accord with those used in Figure 2. “/”: not observed.

Peptide Name	Peptide Name and Sequence	Calculated Exact Mass (Da)	M + H^+^ (Da)	Experimental M + Na^+^ (Da)	M + K^+^ (Da)
**1C**	AcFAGAFuaGPGCAGA	1056.43	/	1079.58	1095.55
**1H**	AcFAGAFuaGPGHAGA	1090.48	1091.66	1113.67	1129.68
**1K**	AcFAGAFuaGPGKAGA	1081.52	1082.69	1104.69	1120.66
**1R**	AcFAGAFuaGPGRAGA	1109.53	1110.75	1132.71	1148.70
**1S**	AcFAGAFuaGPGSAGA	1040.46	/	1063.65	1079.63
**1Y**	AcFAGAFuaGPGYAGA	1116.49	/	1139.70	1155.69

**Table 2:**
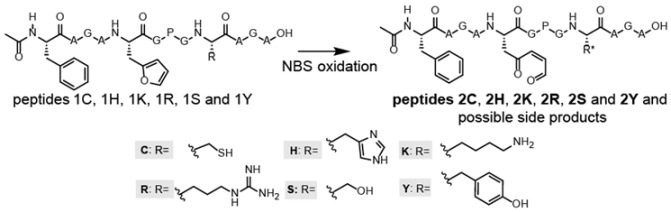
MALDI-TOF analysis of the oxidized template peptides after C-tip reverse phase purification. Peptides 2 and their observed mass values are depicted in bold. “/”: not observed.

Peptides 1 **Peptides 2**	**M + H^+^**	**M + Na^+^**	**M + K^+^**	**Other**
1C **2C**	/	/	/	1214.97 (K^+^ + cysteinylation) **1230.97** (K^+^ + cysteinylation + 16)
1H **2H**	/	/	1130.18 **1146.21**	/
1K **2K**	1082.85	1105.13	1121.06 **1137.09**	**1062.11 (M + H − 20 Da)**
1R **2R**	1111.13 **1127.17**	/	1149.17	/
1S **2S**	/	1064.06	1080.00 **1096.00**	**1021.43 (M + H − 20 Da)**
1Y **2Y**	/	/	/	1313.91 (dibromination) **1329.87** (dibromination + 16)

## Supplementary Materials

The supplementary materials are available online at http://www.mdpi.com/2227-9059/6/4/99/s1.
