# Supplementary material for: Pyrrole-Mediated Peptide Cyclization Identified through Genetically Reprogrammed Peptide Synthesis
Source: Biomedicines. 2018 Oct 30;6(4):99. doi: 10.3390/biomedicines6040099 (PMC6315747; doi:10.3390/biomedicines6040099)
Supplement: Supplementary file 1 [file biomedicines-06-00099-s001.pdf]

SUPPORTING INFORMATION

Pyrrole-mediated peptide cyclization identified through genetically reprogrammed peptide synthesis

Klaas W. Decoene [1], Willem Vannecke[1], Toby Passioura [2], Hiroaki Suga[2] and Annemieke Madder [1,*]

1 Department of Organic and Macromolecular Chemistry, Ghent University, Krijgslaan 281 S4, 9000 Ghent, Belgium; annemieke.madder@ugent.be

2 Department of Chemistry, Graduate School of Science, The University of Tokyo, 7-3-1 Bunkyo-ku, Tokyo 113-0033, Japan; toby@chem.s.u-tokyo.ac.jp

* Correspondence: annemieke.madder@ugent.be

Content:

1. **Furylalanine-CME synthesis**
2. ***N*-acetylphenylalanine synthesis**
3. **DNA template**
4. **Peptide library MALDI-TOF spectra**
5. **NBS oxidation MALDI-TOF spectra**
6. **SPPS peptides (1K, 1S, 1G)**
7. **Peptide NBS oxidation: 2K, 2S, 2G**
8. **One pot oxidation and reduction**
9. **SPPS of template variations 4K and 5K**
10. **One-pot oxidation and reduction 6K, 7K**
11. **NMR analysis of 6K**
12. **Catalogue numbers of products and instruments**

1. Furylalanine-CME synthesis

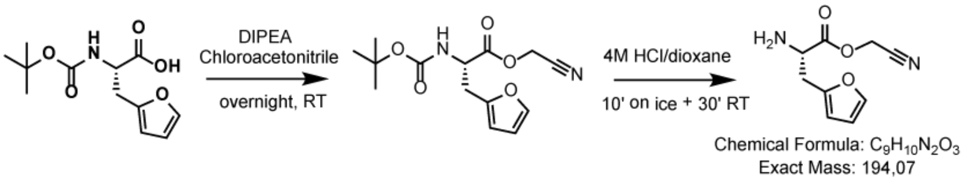

Figure S1.1. The synthesis scheme for the synthesis of furylalanine-CME.

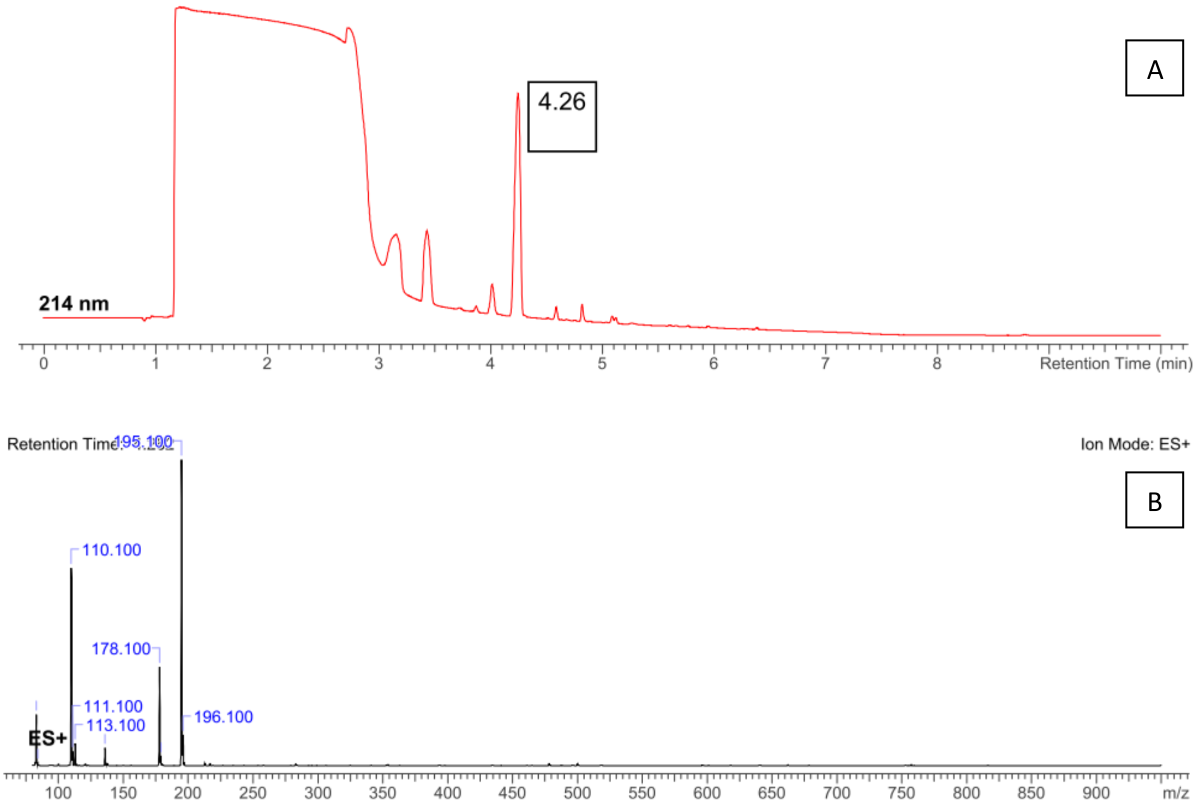

Figure S1.2. A. LCMS chromatogram showing the furylalanine-CME product at t_r= 4.26 min (Kinetex). **B**. ESI-MS from LCMS at t_r= 4.26 min ($M+H^+$ = 195.10 Da)

The synthesis is described in the experimental part of the article.

2. N-acetylated phenylalanine-CME synthesis

Synthesized as previously reported in Goto et al (2006).

3. DNA template

The sequence of the translated template peptide **1** was AcFAGAFuaGPGXAGA (with **X** = C, H, K, R, S, Y). Both residues indicated in red were incorporated in response to the ATG codon.

Forward primer (5' to 3'):
TAATACGACTCACTATAGGGTTAACTTTAACAAGGAGAAAAACATG

Reverse primer (5' to 3'):
GCTAGCTTAAGCACCCGC

Final DNA: Coding sequence + 5' adaptor:

1C CAAGGAGAAAAACATGGCCGGTGCGATGGGTCCGGGCTGCGCGGGTGCTTAA

1H CAAGGAGAAAAACATGGCCGGTGCGATGGGTCCGGGCCACGCGGGTGCTTAA

1K CAAGGAGAAAAACATGGCCGGTGCGATGGGTCCGGGCAAGGCGGGTGCTTAA

1R CAAGGAGAAAAACATGGCCGGTGCGATGGGTCCGGGCCGCGCGGGTGCTTAA

1S CAAGGAGAAAAACATGGCCGGTGCGATGGGTCCGGGCAGCGCGGGTGCTTAA

1K CAAGGAGAAAAACATGGCCGGTGCGATGGGTCCGGGCTACGCGGGTGCTTAA

The template was amplified by PCR. Master mix (1 mL) was prepared by adding 100 µL PCR buffer (10 x) to 820 µL MQ H_2O, 10 µL $MgCl_2$ 250 mM, 50 µL5 mM of each dNTP mix, 5 µL 100 µM forward primer, 5 µL 100 µM reverse primer and 10 µL (100 x) Taq polymerase. This master mix was divided in 6 PCR tubes and different test oligo's were added (0.5 µL 1 µM solution) to each tube. The PCR reaction was set up: 95 °C 40 sec., 50 °C 40 sec., 72°C 40 sec. and repeated for 15 cycles. And agarose gel was run with 2µL sample mixed with 2 µL of 2x DNA loading buffer. The gel was a 3% agarose gel run at 135 V for 10 min.

4. Peptide library MALDI-TOF spectra

Template peptide **1**: AcFAGAFuaGPGXAGA (**1C**:X=C, **1H**:X=H, **1K**:X=K, **1R:X**=R, **1S**:X=S, **1Y**:X=Y)

Peptide 1C

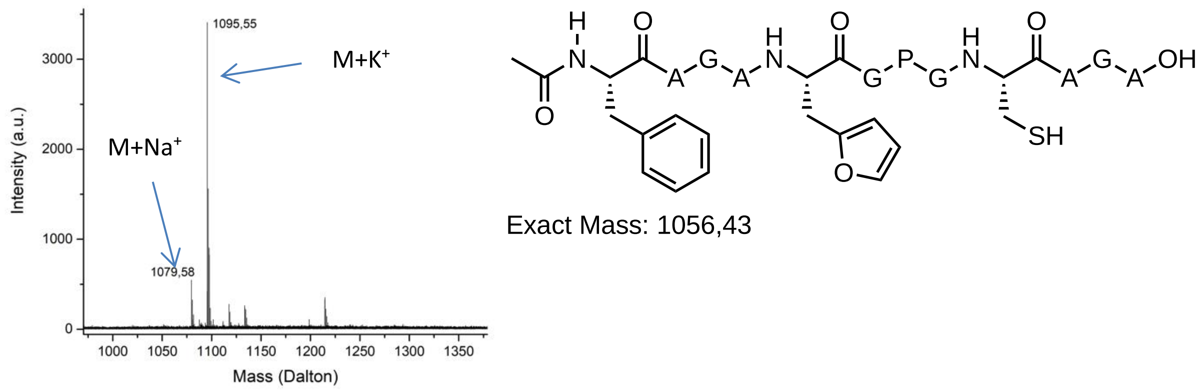

Figure S4.1. MALDI-TOF spectrum of translated peptide **1C** and the structure with exact mass. The M+K^{+} peak: 1056.43 Da + 39.10 Da = 1095.53 Da and M+Na^{+} peak:1056.43 Da + 22.99 Da = 1079.42 Da peak are indicated with arrows in the mass spectrum.

Peptide 1H

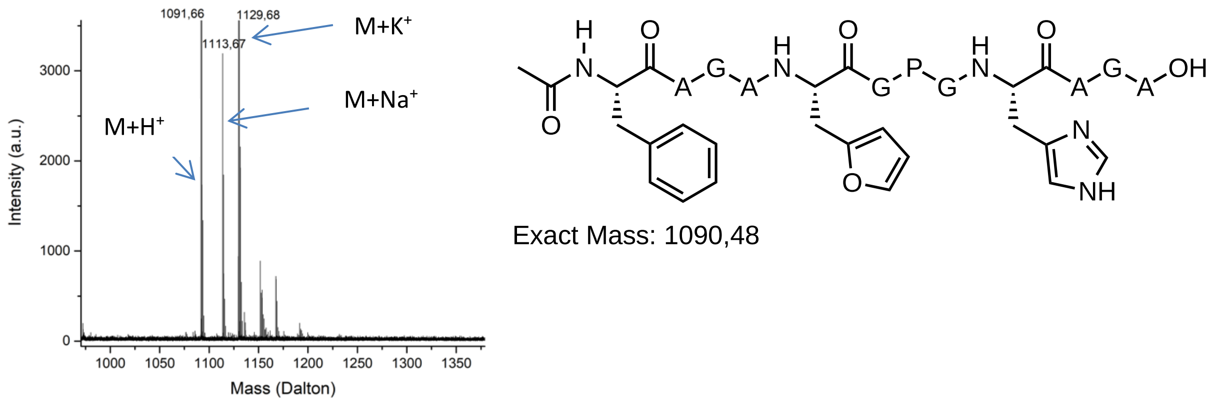

Figure S4.2. MALDI-TOF spectrum of translated peptide **1H** and the structure with exact mass. The M+H^{+} peak: 1090.48 Da + 1.01 Da = 1091.49, M+K^{+}peak : 1090.48 Da + 39.10 Da = 1129.58 Da and M+Na^{+} peak :1090.48 Da + 22.99 Da = 1113.47 Da are indicated with arrows in the mass spectrum.

Peptide 1K

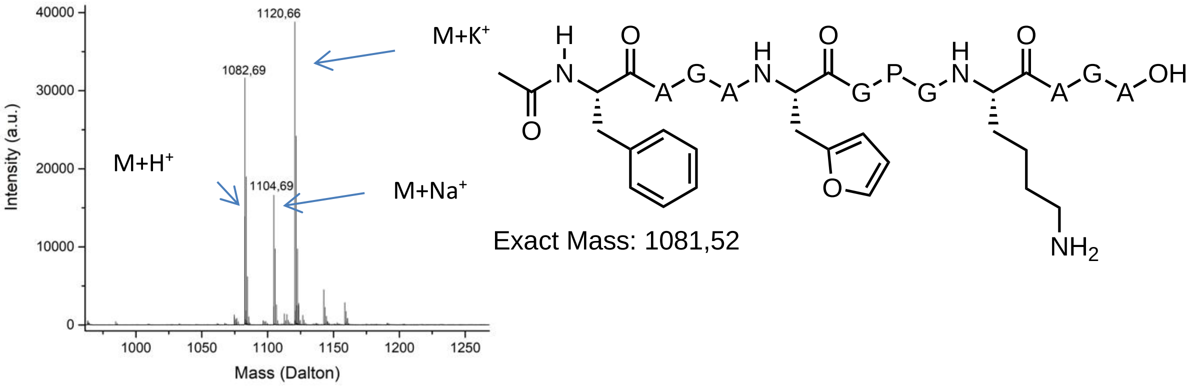

Figure S4.3. MALDI-TOF spectrum of translated peptide **1K** and the structure with exact mass. The M+H$^+$ peak: 1081.52 Da + 1.01 Da = 1082.53, M+K$^+$ peak: 1081.52 Da + 39.10 Da = 1120.62 Da and M+Na$^+$ peak: 1081.52 Da + 22.99 Da = 1104.51 Da are indicated with arrows in the mass spectrum.

Peptide 1R

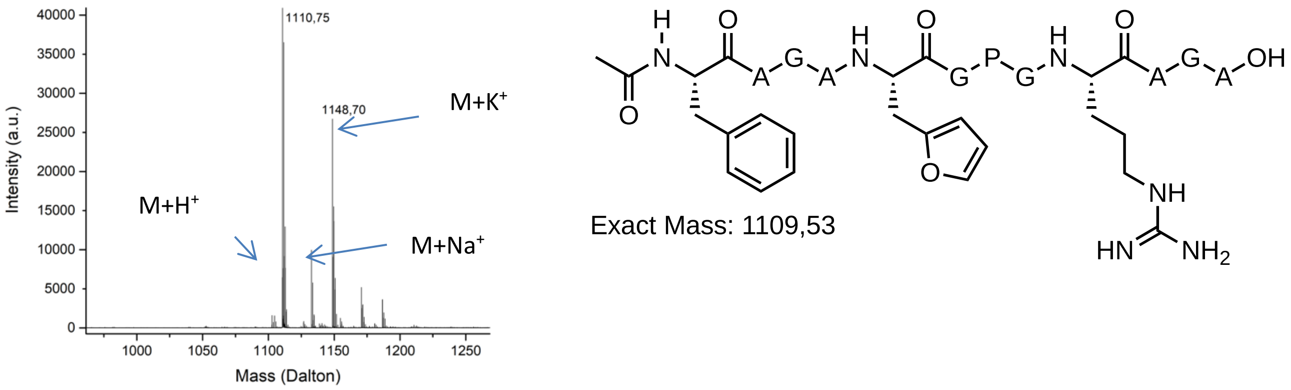

Figure S4.4. MALDI-TOF spectrum of translated peptide **1R** and the structure with exact mass. . The M+H$^+$ peak: 1109.53 Da + 1.01 Da = 1110.54, M+K$^+$ peak: 1109.53 Da + 39.10 Da = 1148.63 Da and M+Na$^+$ peak :1109.53 Da + 22.99 Da = 1132.52 Da are indicated with arrows in the mass spectrum.

Peptide 1S

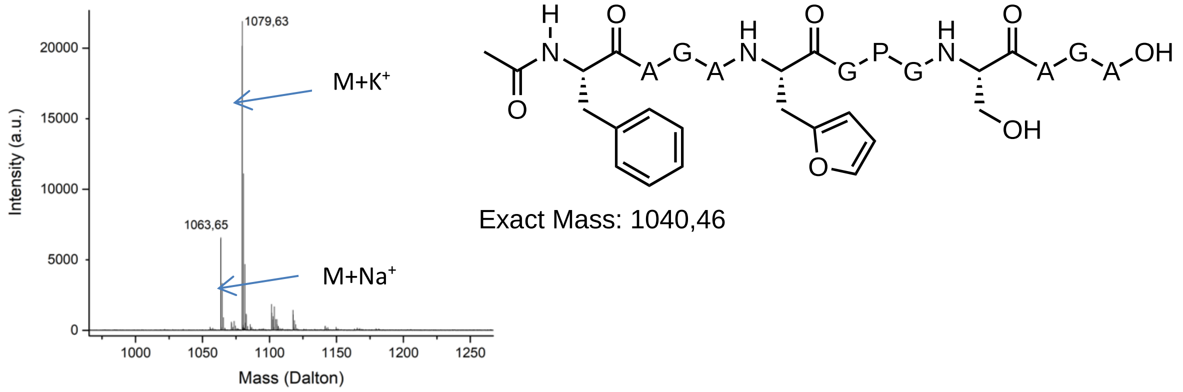

Figure S4.5. MALDI-TOF spectrum of translated peptide **1S** and the structure with exact mass. The $M+K^{+}$ peak: 1040.46 Da + 39.10 Da = 1079.56 Da and $M+Na^{+}$ peak: 1040.46 Da + 22.99 Da = 1063.45 Da are indicated with arrows in the mass spectrum.

Peptide 1Y

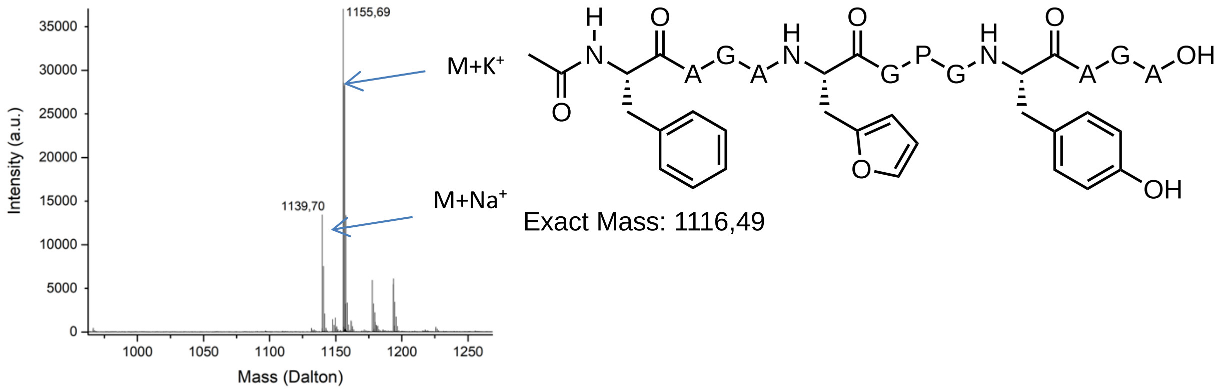

Figure S4.6. MALDI-TOF spectrum of translated peptide **1Y** and the structure with exact mass. The $M+K^{+}$ peak : 1116.49 Da + 39.10 Da = 1155.59 Da and $M+Na^{+}$ peak :1116.49 Da + 22.99 Da = 1139.48 Da are indicated with arrows in the mass spectrum.

5. NBS oxidation MALDI-TOF spectra

For the oxidation of the translated peptides, 2.5 µL of translation mixture was used and 0.5 µL of a 1:1 (saturated NBS in H_2O: H_2O) was added. Note that it is not possible to measure the amount of peptide present in the translated mixture and that there are a multitude of other proteins (needed for translation) present. Samples were analysed via MALDI-TOF after C-tip purification.

Peptide 2C

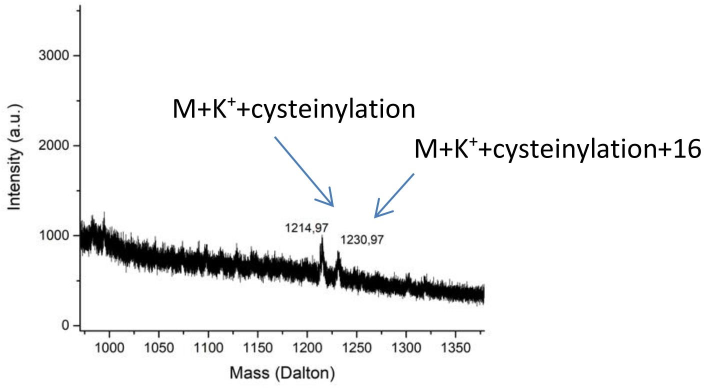

Figure S5.1. MALDI-TOF spectrum of translated and NBS oxidized peptide **2C**. The cysteinylation peak: 1056.43 Da + 39.10 Da + 119 Da = 1214.54 Da and the oxidized cysteinylation peak: 1056.43 Da + 39.10 Da + 119 Da + 16 Da = 1230.54 Da are indicated in the spectrum.

Peptide 2H

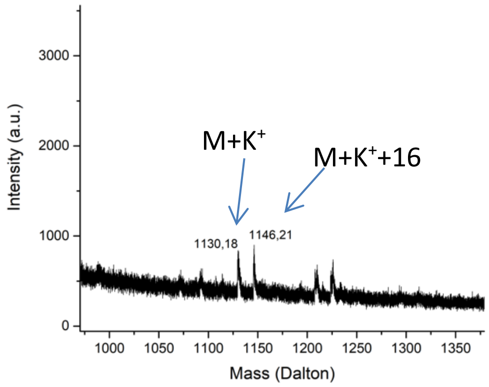

Figure S5.2. MALDI-TOF spectrum of translated and NBS oxidized peptide **2H**.The M+K$^+$ peak: 1090.48 Da + 39.10 Da = 1129.20 Da and M+K$^+$+16 peak: 1090.48 Da + 39.10 Da + 16 Da = 1145.58 Da are indicated in the spectrum.

Peptide 2K

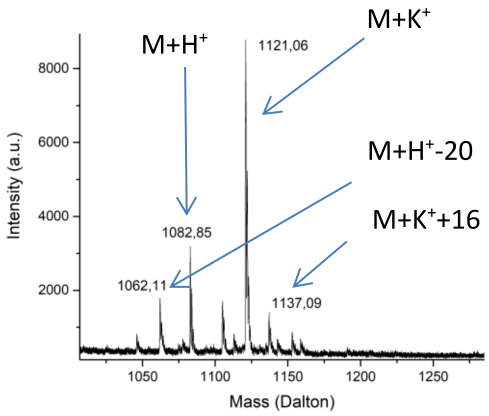

Figure S5.3. MALDI-TOF spectrum of translated and NBS oxidized peptide **2K** . The $M+H^{+}$-20 peak: 1081.52 Da + 1.01 Da -20 Da = 1062.53 Da, $M+H^{+}$ peak: 1081.52 Da + 1.01 Da = 1082.53 Da, $M+K^{+}$ peak: 1081.52 Da + 39.10 Da = 1120.62 Da, $M+K^{+}$+16 peak: 1081.52 Da + 39.10 Da +16 Da = 1136.62 Da are indicated in the spectrum.

Peptide 2R

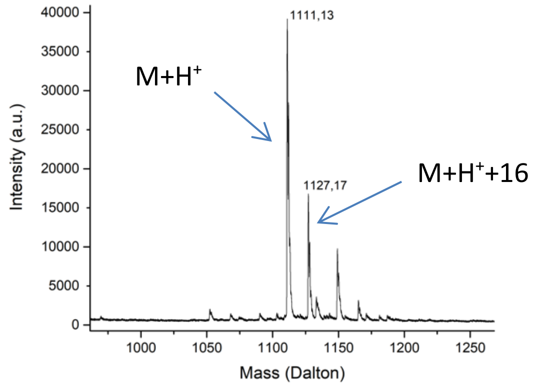

Figure S5.4. MALDI-TOF spectrum of translated and NBS oxidized peptide **2R** . $M+H^{+}$ peak: 1109.53 Da + 1.01 Da = 1110.54 Da, $M+H^{+}$+16 peak: 1109.53 Da + 1.01 Da +16 Da = 1126.62 Da are indicated in the spectrum.

Peptide 2S

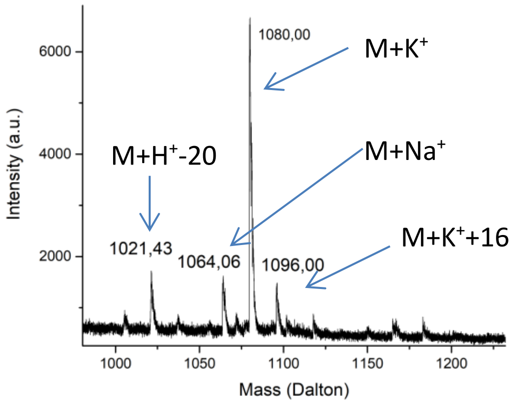

Figure S5.5. MALDI-TOF spectrum of translated and NBS oxidized peptide **2S**. The M+H$^+$-20 peak: 1040.46 Da + 1.01 Da -20 Da = 1021.47 Da, M+H$^+$ peak: 1040.46 Da + 1.01 Da = 1041.47 Da, M+K$^+$ peak: 1040.46 Da + 39.10 Da = 1079.56 Da, M+K$^+$+16 peak: 1040.46 Da + 39.10 Da +16 Da = 1095.56 Da are indicated in the spectrum.

Peptide 2Y

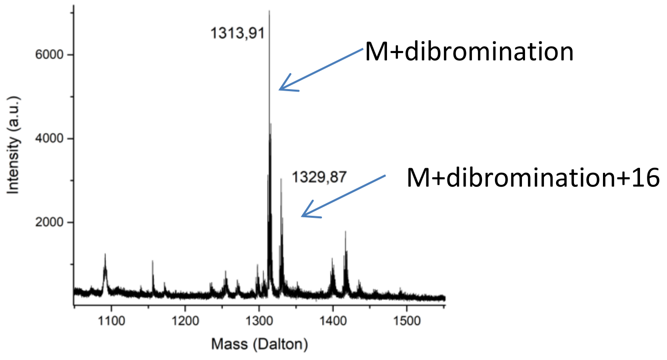

Figure S5.6. MALDI-TOF spectrum of translated and NBS oxidized peptide **2Y**. The dibromination peak: 1116.49 Da + 158 Da +39.10 Da = 1313.59 Da and the oxidized dibromination peak: 1116.49 Da + 158 Da + 39.10 Da + 16 Da = 1329.59 Da are indicated in the spectrum.

6. SPPS of peptides 1K, 1S, 1G

Peptides were synthesized on an Intavis automated peptide synthesizer as described in the experimental section and purified on a HPLC Luna semiprep column (0-100 ACN in 20 min).

Peptide 1K

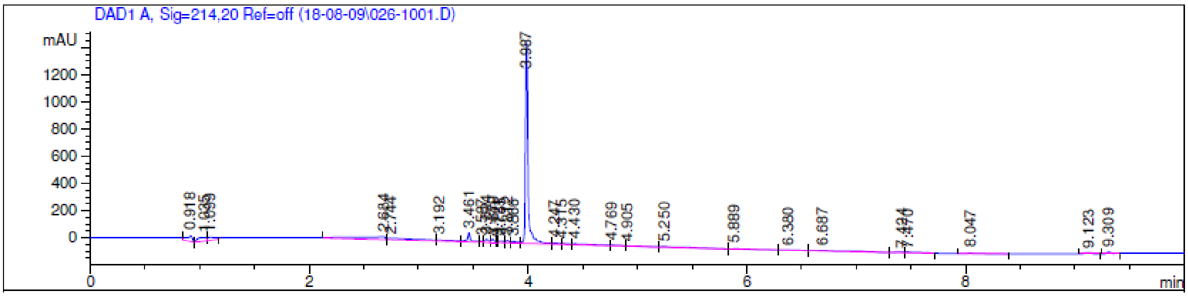

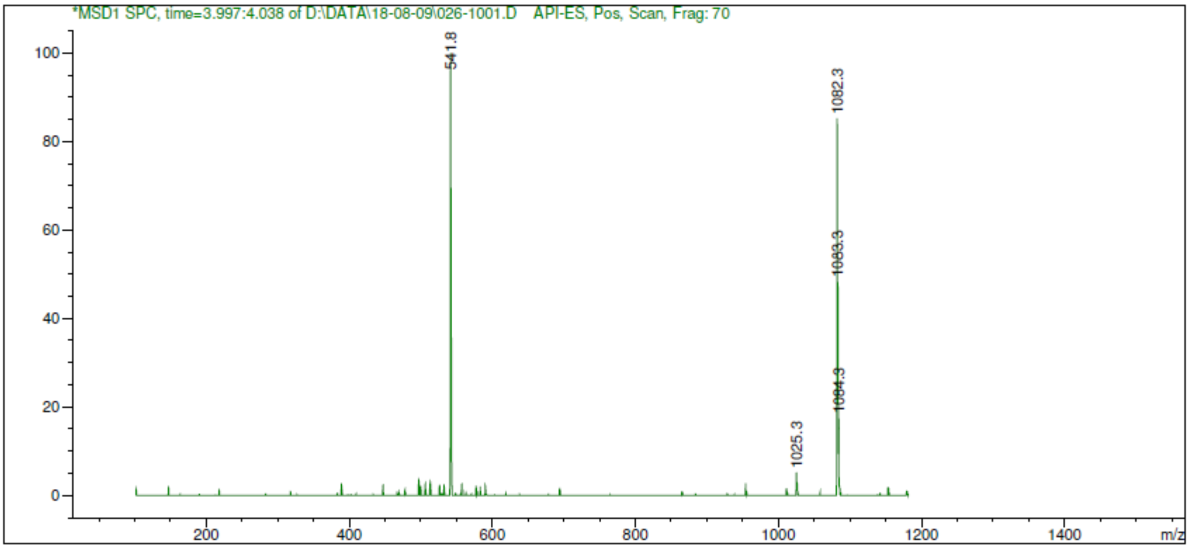

Figure S6.1. LCMS analysis of **1K** (Exact mass = 1081.5), top: LC 214 nm chromatogram, bottom: ESI-MS (positive mode) spectrum.

Peptide 1S

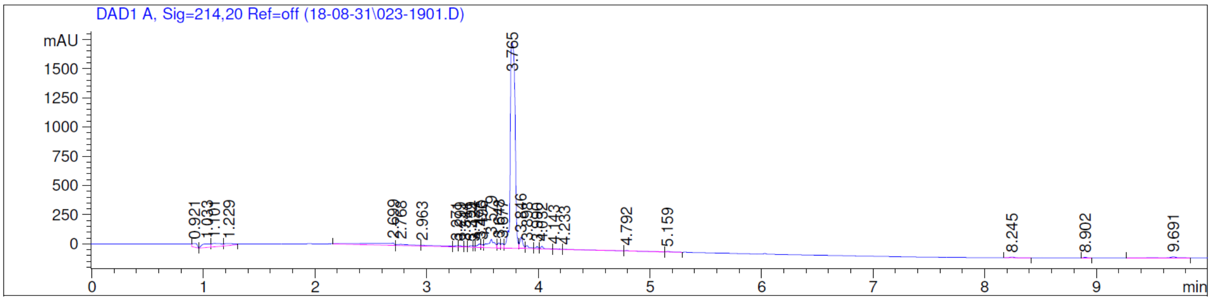

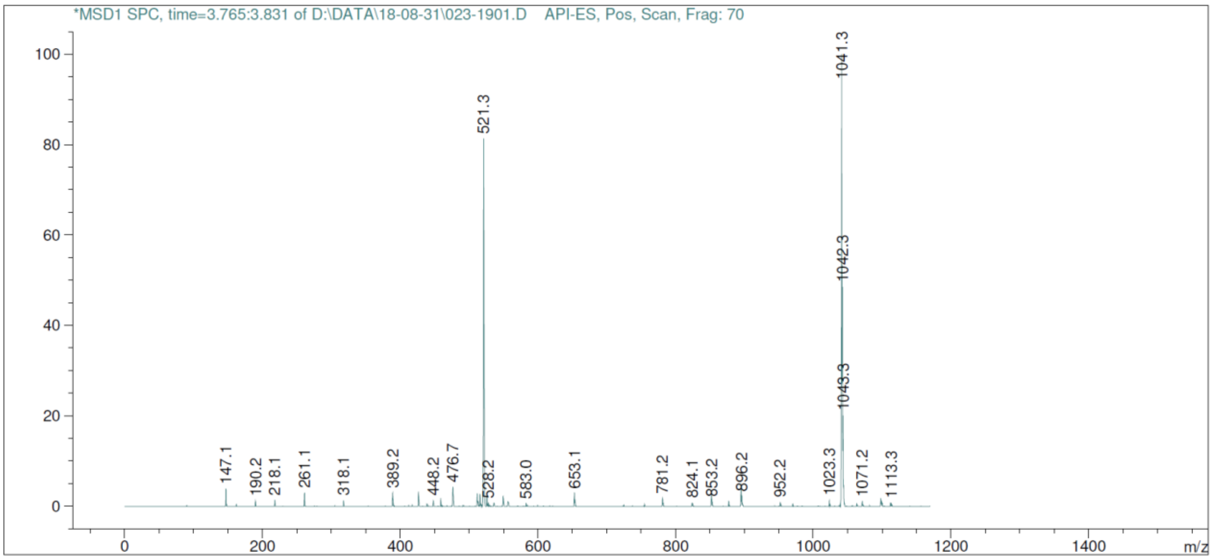

Figure S6.2. LCMS analysis of **1S** (Exact mass = 1040.5), top: LC 214 nm chromatogram, bottom: ESI-MS (positive mode) spectrum.

Peptide 1G

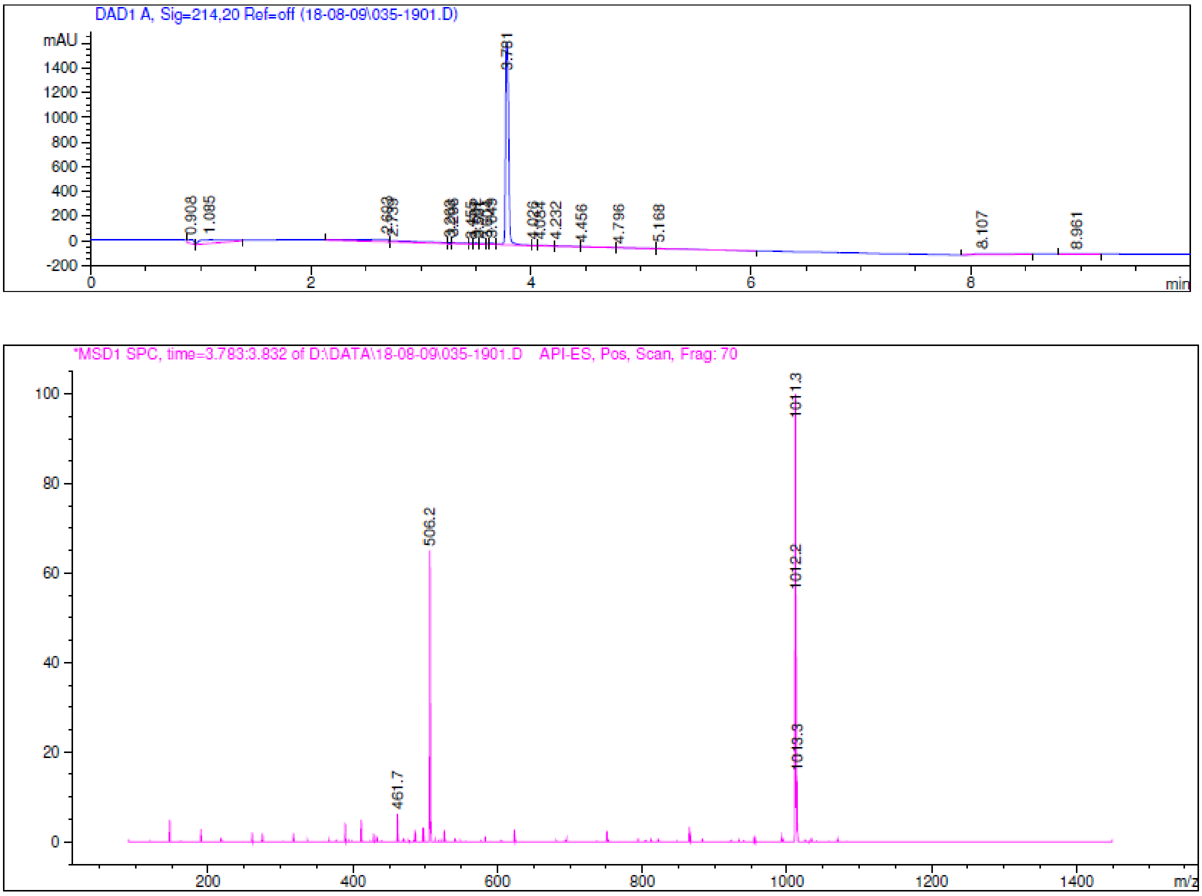

Figure S6.3. LCMS analysis of **1G** (Exact mass = 1010.5), top: LC 214 nm chromatogram, bottom: ESI-MS (positive mode) spectrum.

7. Peptide NBS oxidation towards 2K, 2S and 2G

Peptides were solubilized in MQ H_2O with a concentration of 2.5 mM. 10 µL of this peptide solution was added to 10 µL NaOAc buffer (pH 5.2) and 3 eq of NBS were added for the oxidation reaction. In the case of **2S** the amount of peptide used was higher and this resulted in incomplete peptide oxidation. In all three cases, the MS spectrum of the resulting oxidized product, shows a mass which corresponds to $M+H^{+}$-2 Da. This can be caused by the oxidation reaction (+16 Da) followed by a subsequent loss of water (-18 Da) during either cyclisation or ionisation. To investigate this in more detail, we compared the extracted ion chromatograms of the $M+H^{+}$-2 and $M+H^{+}$+16 ions. For peptides **2G** as well as **2S**, the chromatograms indicate that both ions are resulting from the same compound, while for **2K** it is clear that the $M+H^{+}$-2 ion results from another compound with a slightly different retention time (compare green versus pink traces in the XIC's). This is consistent with the amine side chain of the lysine residue engaging in imine formation with the oxidized furan moiety leading to a mass loss of -2 Da. The LCMS data in the main text of the article consists of zooms of the relevant region. When the oxidation experiments are carried out in presence of NaOAc buffer, 2 large peaks can consistently be found in the chromatogram at 214 nm. Those peaks (marked *) are present in all experiments and are not peptide related.

NBS oxidation of peptide 1G to 2G

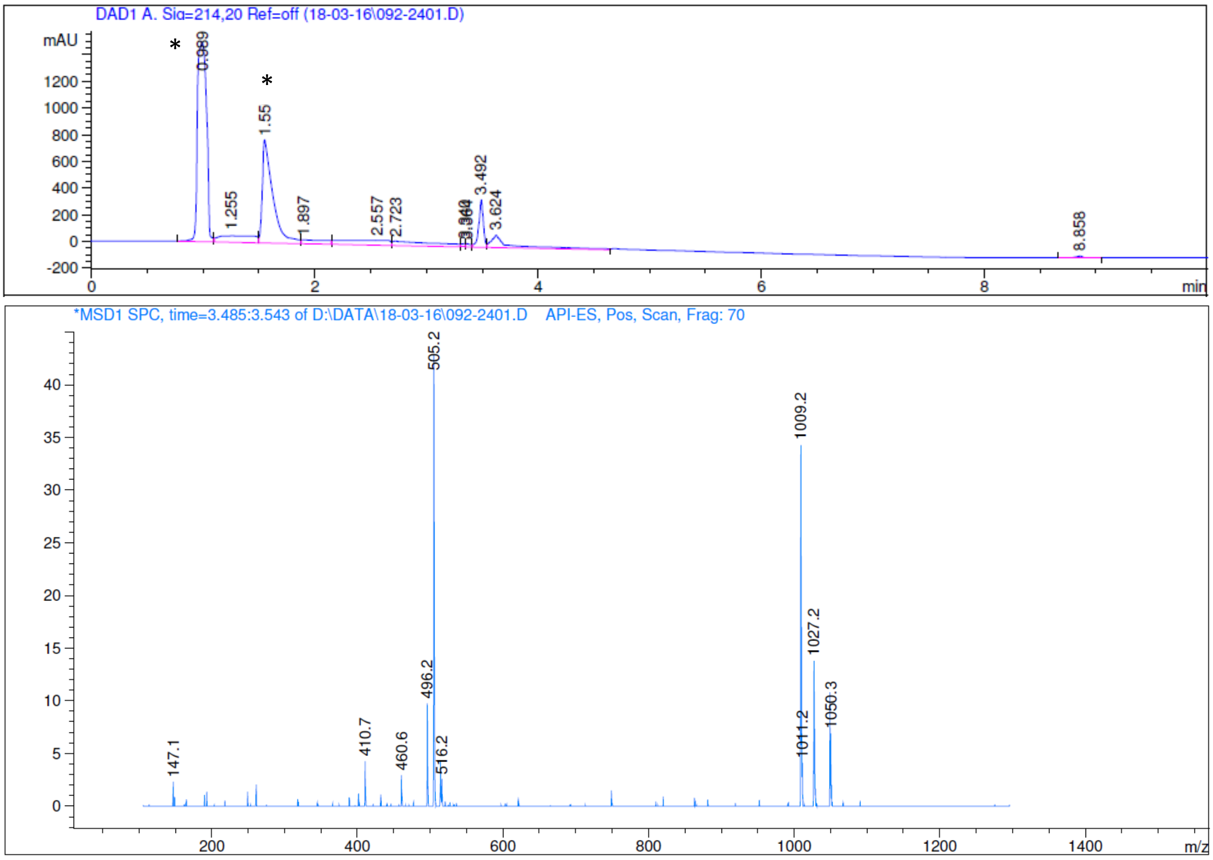

Figure S7.1. LCMS analysis after oxidation of peptide **1G** (exact mass=1010.5) to **2G**, top: LC 214 nm chromatogram, bottom: ESI-MS (positive mode) spectrum of the peak at 3.492 min (resulting from oxidation).

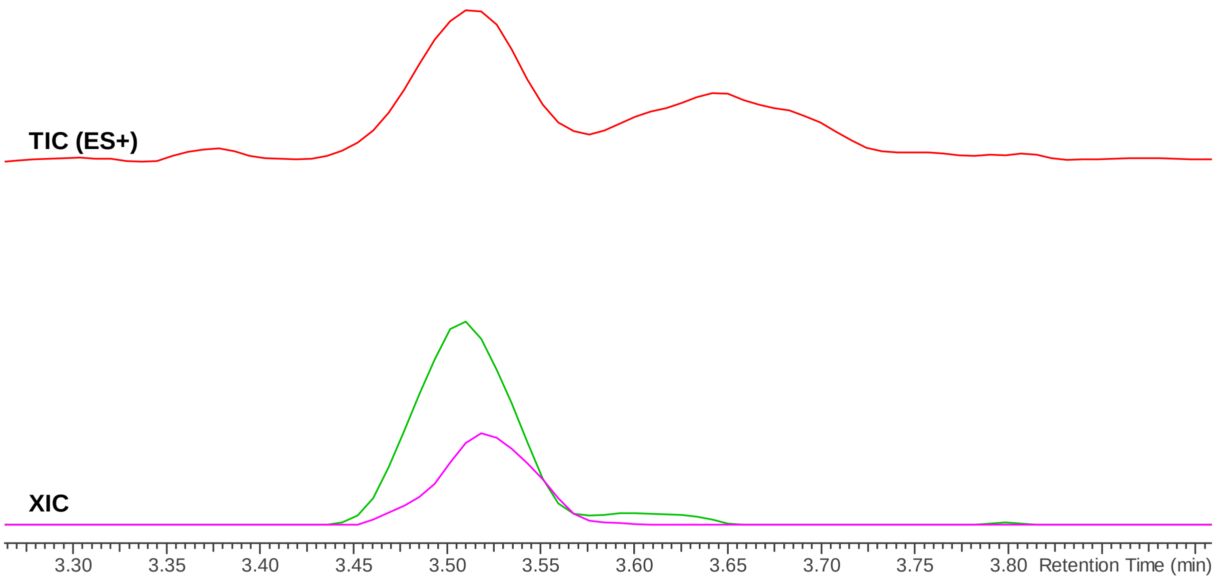

Figure S7.2 TIC (ES+) red and extracted ion counts (green = M+H-2 (1009.20); pink = M+H+16 (1027.20))

NBS oxidation of peptide 1K to 2K

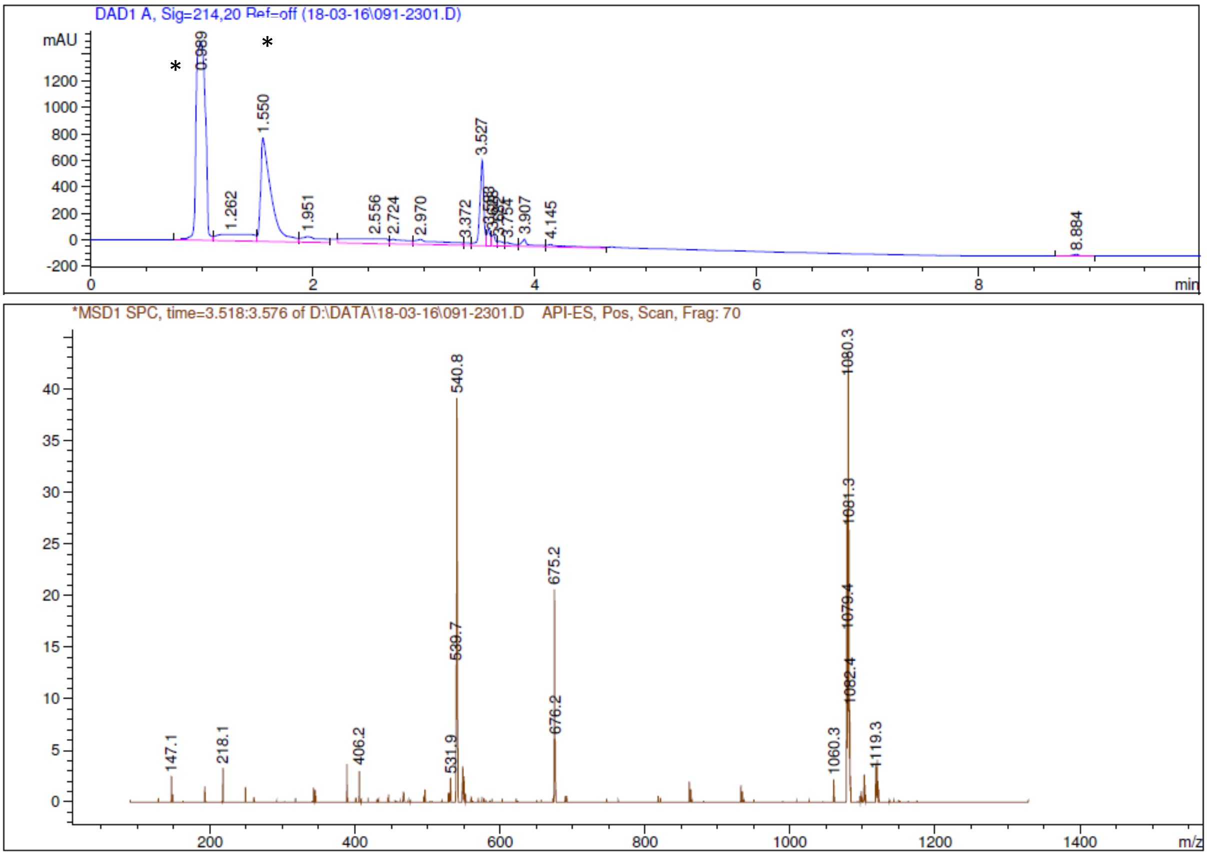

Figure S7.3. LCMS analysis after oxidation of peptide **1K** (Exact mass = 1081.5) to **2K**, top: LC 214 nm chromatogram, bottom: ESI-MS (positive mode) spectrum of the peak at 3.527 min (resulting from oxidation).

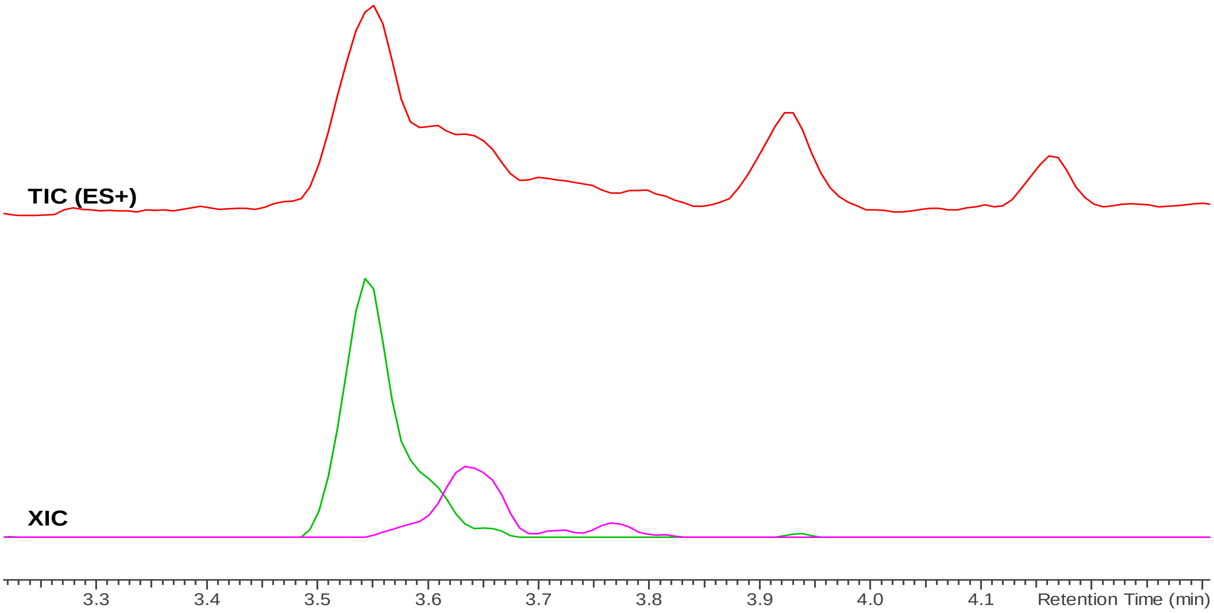

Figure S7.4. TIC (ES+) and extracted ion counts (green = M+H-2 (1080.45); pink = M+H+16 (1098.45))

NBS oxidation of peptide 1S to 2S

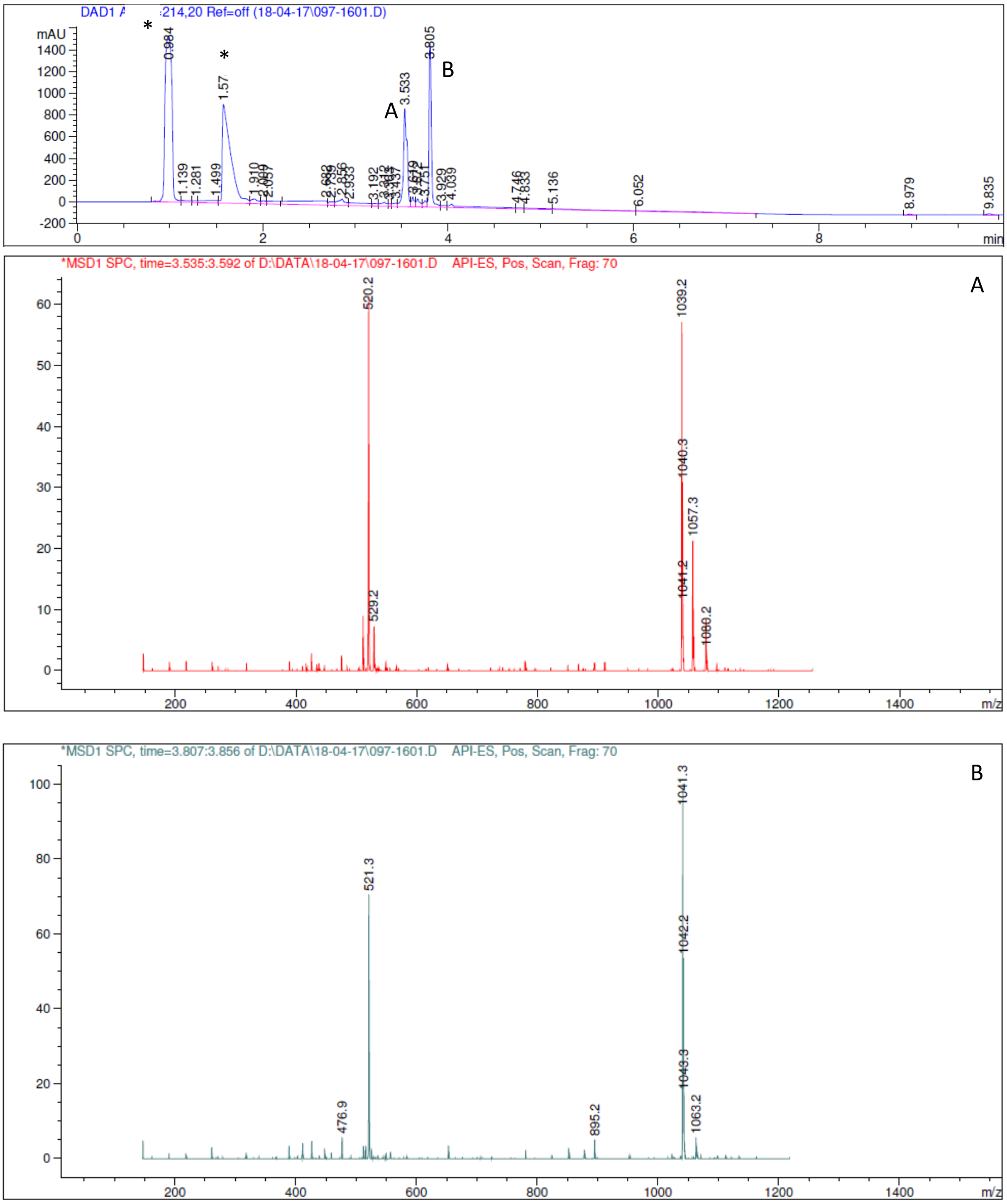

Figure S7.5. LCMS analysis after oxidation of **1S** to **2S** (Exact mass = 1040.5), top: LC 214 nm chromatogram, bottom: ESI-MS (positive mode) spectrum of the two most prominent peaks (resulting from oxidation (A) as well as starting peptide (B)).

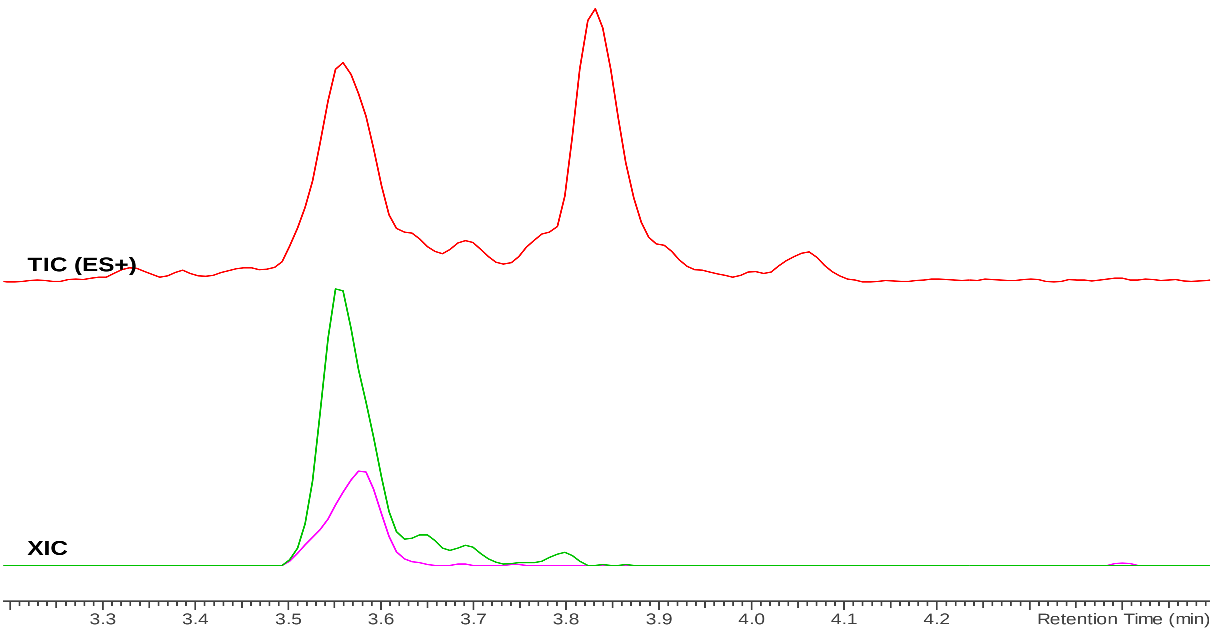

Figure S7.6 TIC (ES+) and extracted ion counts (green = M+H-2 (1039.20); purple = M+H+16 (1057.35))

8. <u>One pot oxidation and reduction</u>

The setup for the one pot oxidation and reduction reactions of peptides **1K**, **1G** and **1S** was very similar to that of the oxidation reaction. The same peptide stock solutions of 2.5 mM were used, and the oxidation reaction was done exactly the same as in 7. Following the addition of NBS, the sample was mixed well and afterwards 100 eq of $NaCNBH_3$ was added to reduce the formed imine. The samples were analysed by LCMS.

<u>Oxidation/reduction of peptide 1K to 3K</u>

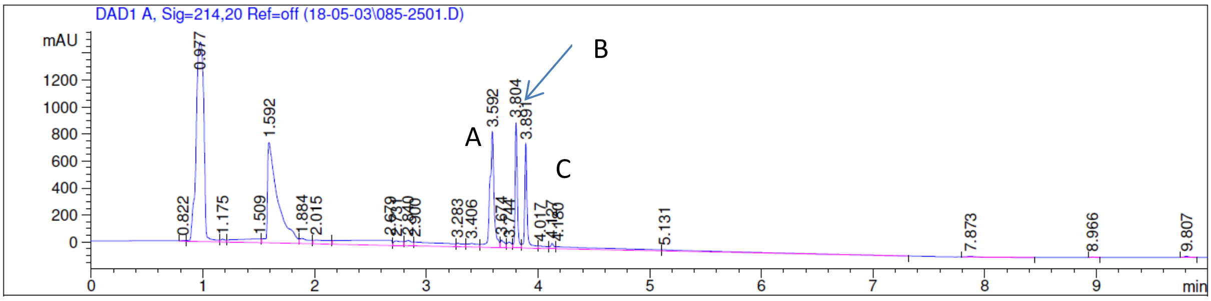

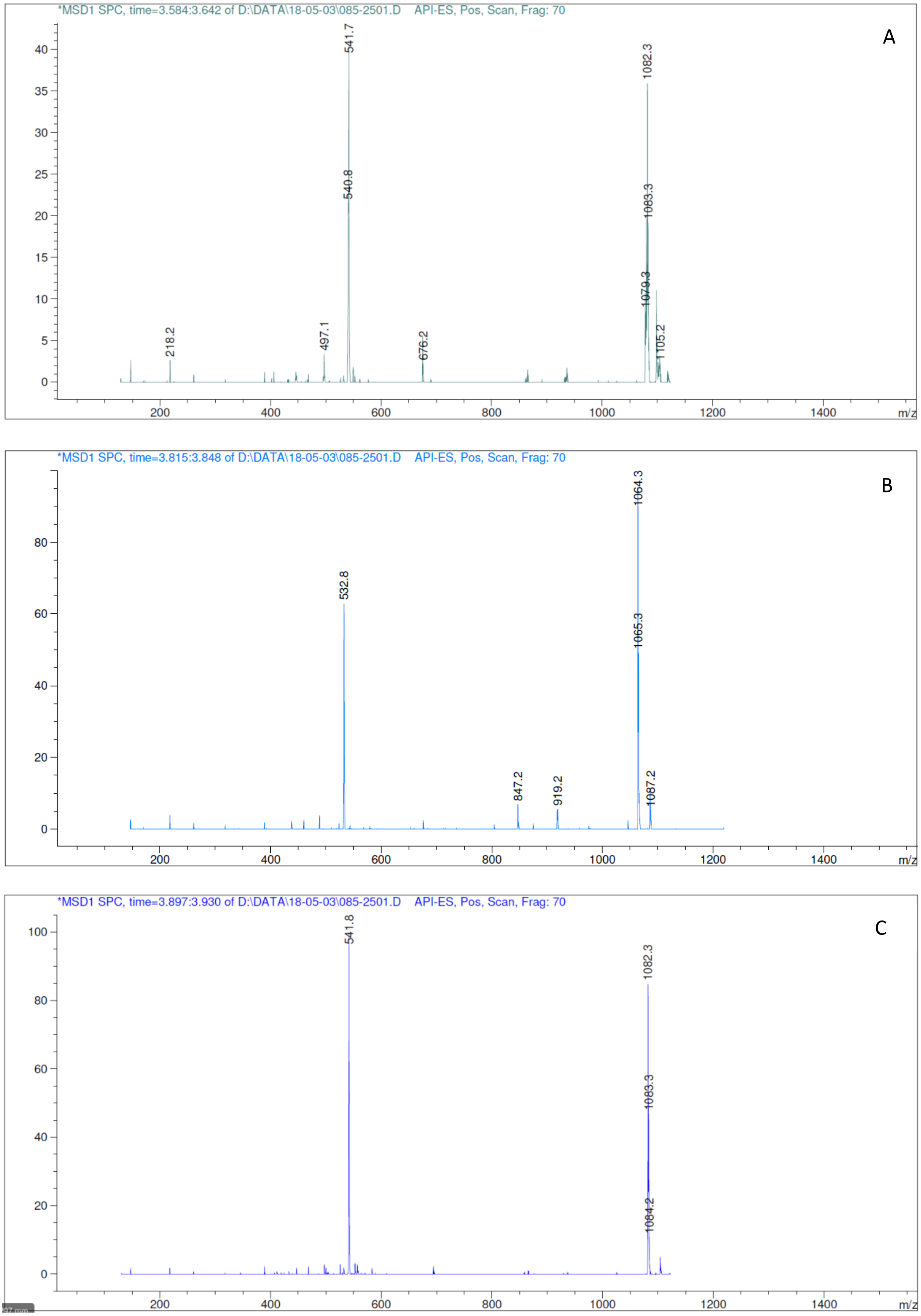

Figure S8.1. LC chromatogram at 214 nm for oxidation / reduction / cyclisation of peptide **1K** (Exact mass = 1081.5) to **3K** and ESI-MS (positive mode) spectra for peaks A, B and C.

Peak A: oxidation + imine formation + reduction + H: 1081.5 + 16 – 18 + 2 + 1 = 1082.5
Peak B: oxidation + imine formation + reduction+ pyrrole formation + H: 1081.5 + 16 – 18 + 2 -18 + 1 = 1064.5
***Peak C*: **starting material with Exact mass = 1081.5

Oxidation/reduction of peptide 1G to 3G

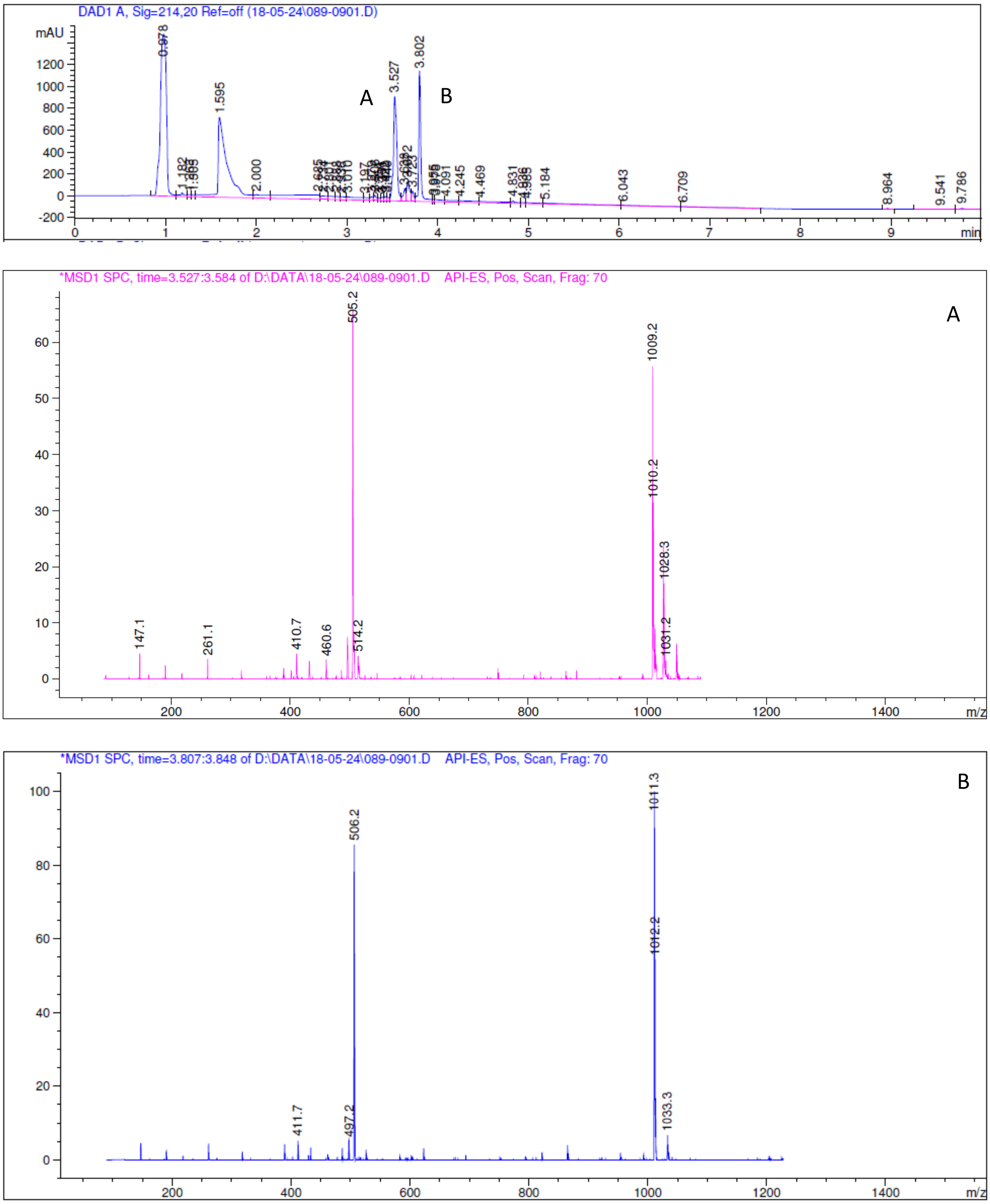

Figure S8.2. LC chromatogram at 214 nm for oxidation / reduction of peptide **1G** (Exact mass = 1010.3) to **3G** and ESI-MS (positive mode) spectra for peaks A and B.

Peak A: oxidation + loss of water during ionisation + H: 1010.5 + 16 – 18 + 1 = 1009.5.
Peak B: starting material with Exact mass = 1010.5.

Oxidation/reduction of peptide 1S to 3S

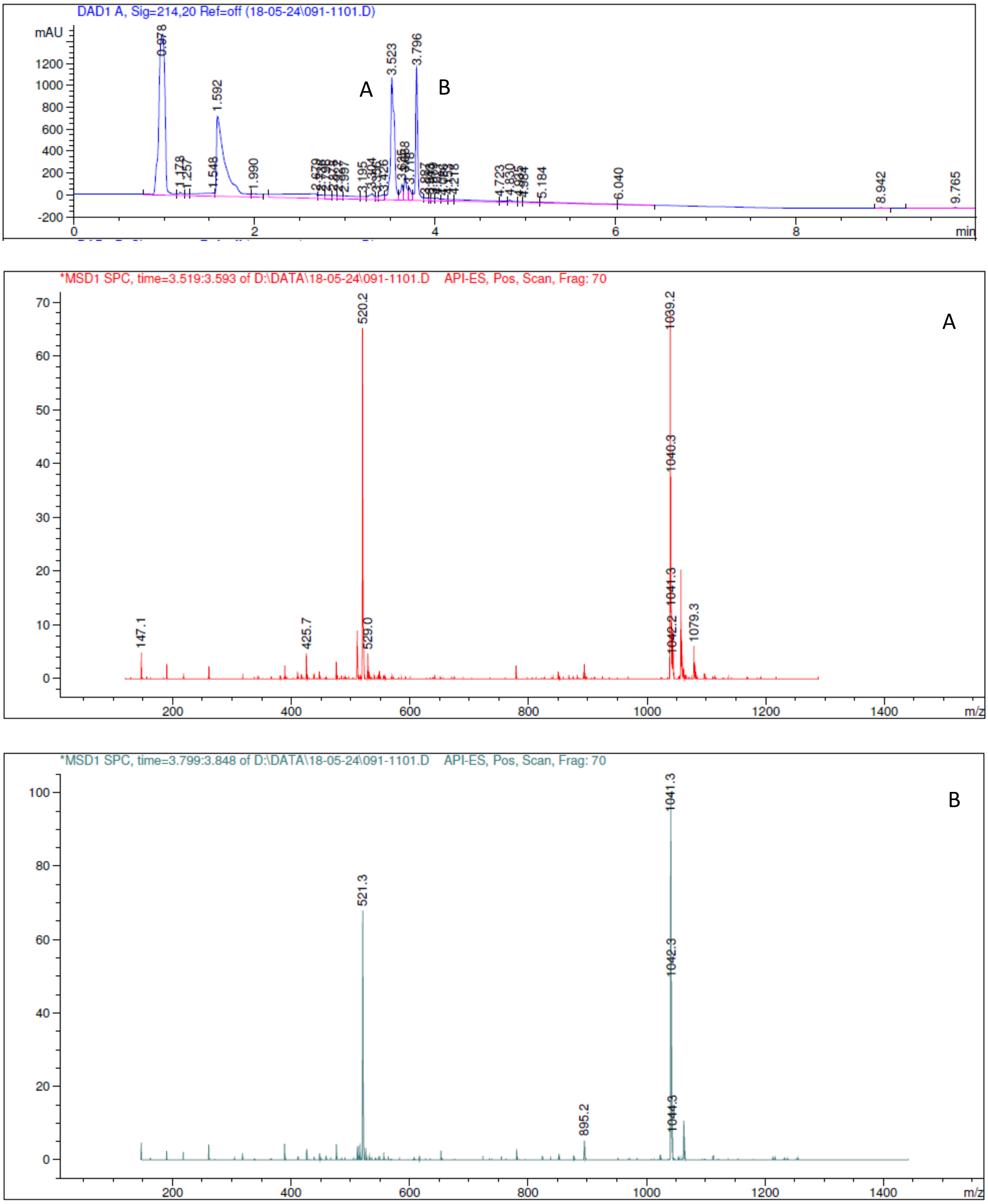

Figure S8.3. LC chromatogram at 214 nm for oxidation / reduction of peptide **1S** (Exact mass = 1040.5) to **3S** spectra for peaks A and B.

Peak A: oxidation + loss of water during ionisation + H: 1040.5 + 16 – 18 + 1 = 1039.5.
Peak B**:** starting material with Exact mass = 1040.5.

9. SPPS of template peptide variations 4K and 5K

Peptides 4K and 5K were synthesized on solid support following the procedure described in the experimental section. The peptides were purified via a prep-HPLC Luna semiprep column (0-100 ACN in 20 min).

Peptide 4K

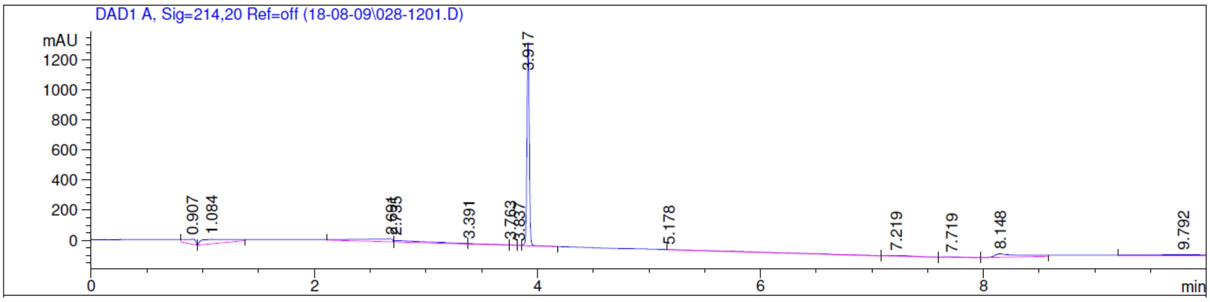

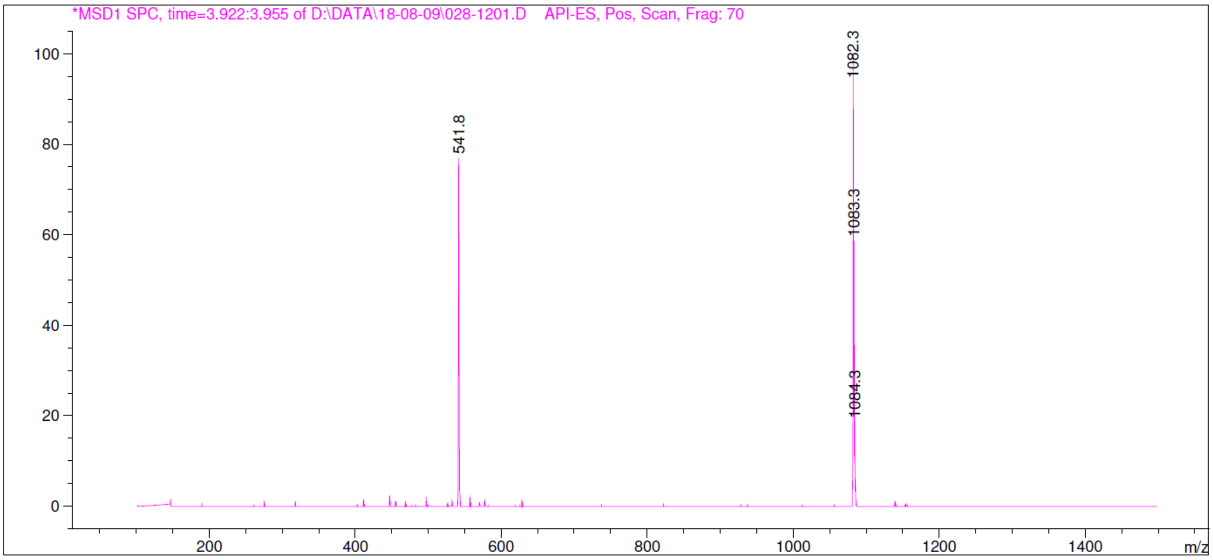

Figure S9.1. LCMS analysis of **4K** (Exact mass = 1081.5), top: LC 214 nm chromatogram, bottom: ESI-MS (positive mode) spectrum.

Peptide 5K

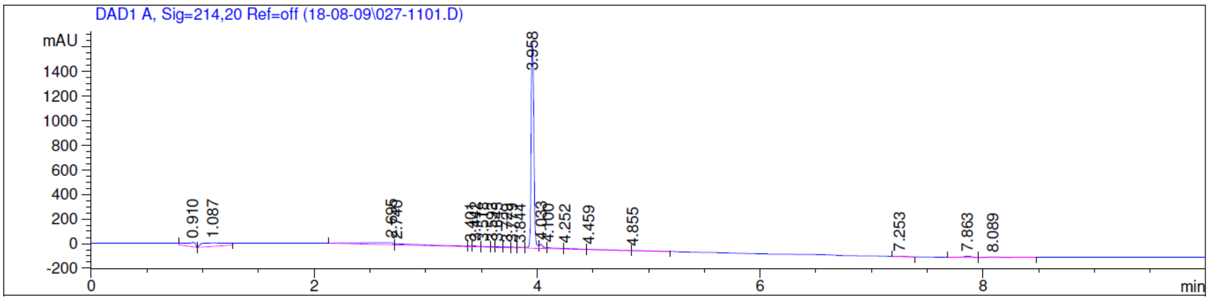

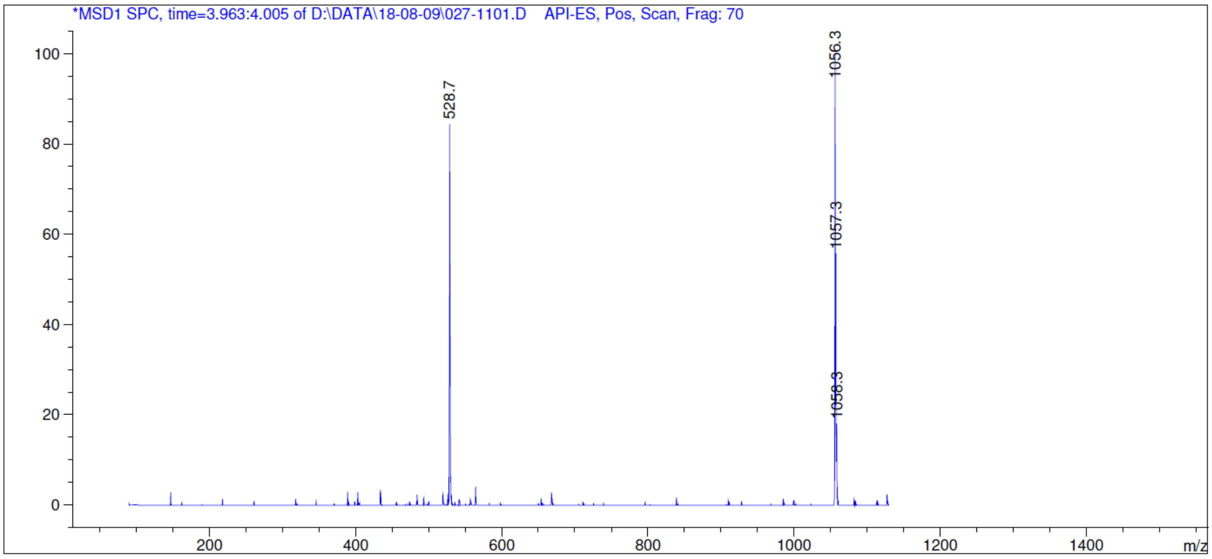

Figure S9.2. LCMS analysis of **5K** (Exact mass = 1055.5), top: LC 214 nm chromatogram, bottom: ESI-MS (positive mode) spectrum.

10. One-pot oxidation and reduction of peptides 4K to 6K and 5K to 7K

The procedure for the one-pot oxidation and reduction reaction was exactly the same as in 8. The peptides were analysed via LCMS

One-pot oxidation and reduction of peptide 4K to 6K

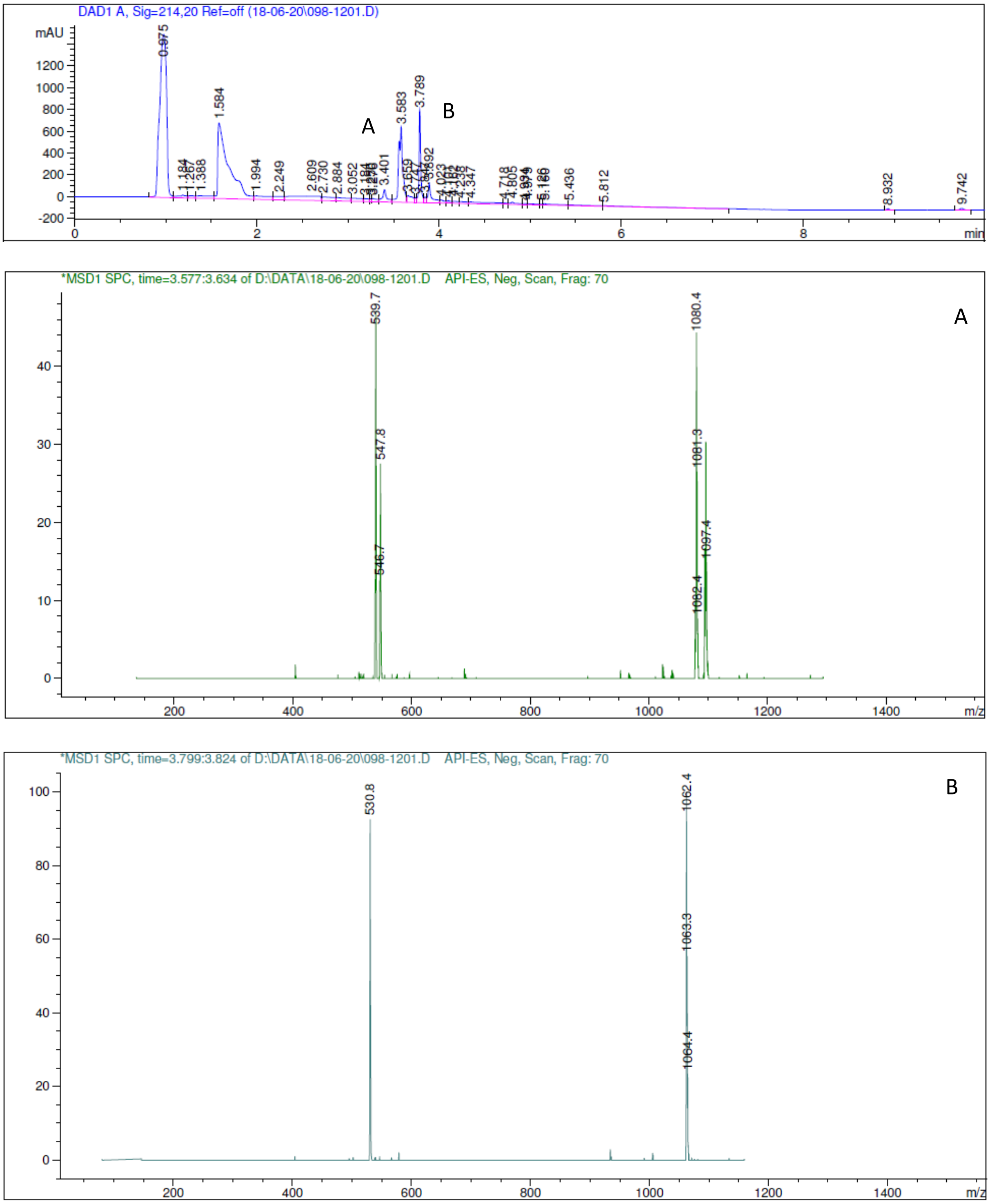

Figure S10.1. LC chromatogram at 214 nm for one pot oxidation / reduction of peptide **4K** (Exact mass = 1081.5) to **6K** and ESI-MS (negative mode) spectra for peaks A and B.

Peak A: a) oxidation - H: 1081.5 + 16 - 1 = 1096.5
b) oxidation + imine formation with loss of water - H: 1081.5 + 16 – 18 + 2 - 1 = 1080.5
Peak B: oxidation + imine formation + reduction + pyrrole formation - H: 1081.5 + 16 – 18 + 2 -18 - 1 = 1062.5

After reaction optimisation it was possible to oxidize almost all of the starting peptide to the oxidized species, in figure S10.2 the starting peptide **4K** had almost completely disappeared. However it seemed that it was not possible to convert all of the oxidized species to the pyrrole compound, in figure S10.2 the peak corresponding to the oxidized products of **4K**. HPLC peak integration demonstrated a conversion of 32 % of the starting compound (**4K**) to the pyrrole constrained peptide **6K**.

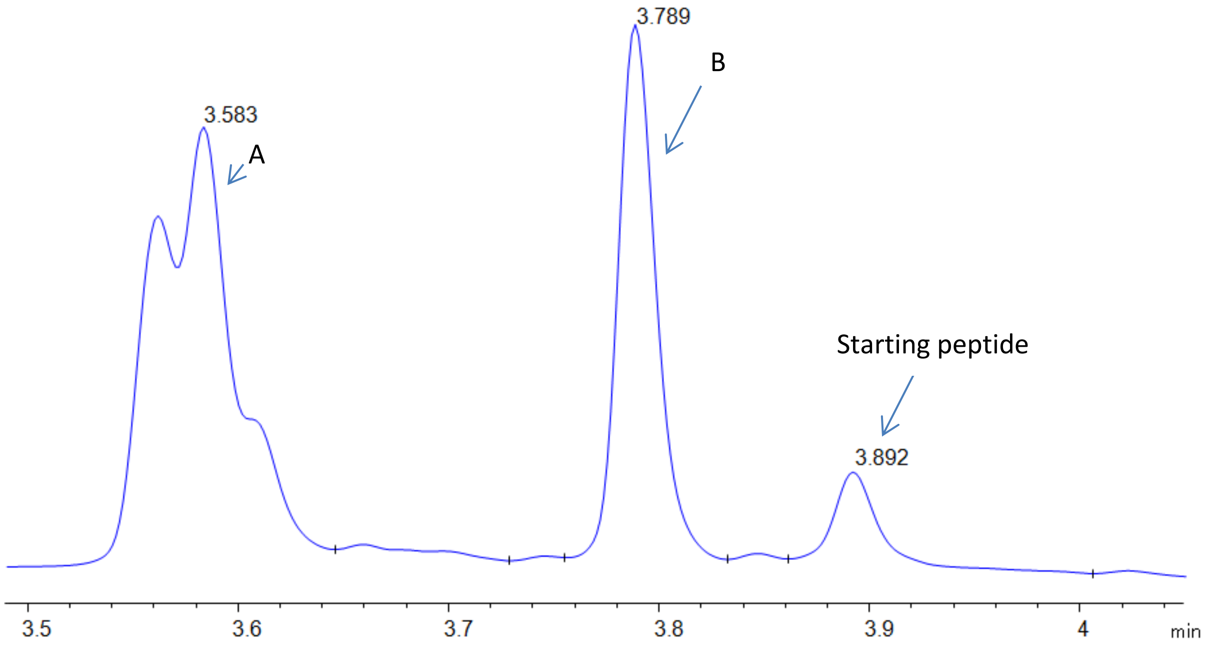

Figure S10.2. Zoom of the LC chromatogram in figure S 10.1. Peaks of the oxidized products (A), pyrrole constrained peptide **6K** (B) and the starting peptide are indicated on the chromatogram.

One-pot oxidation and reduction of peptide 5K to 7K

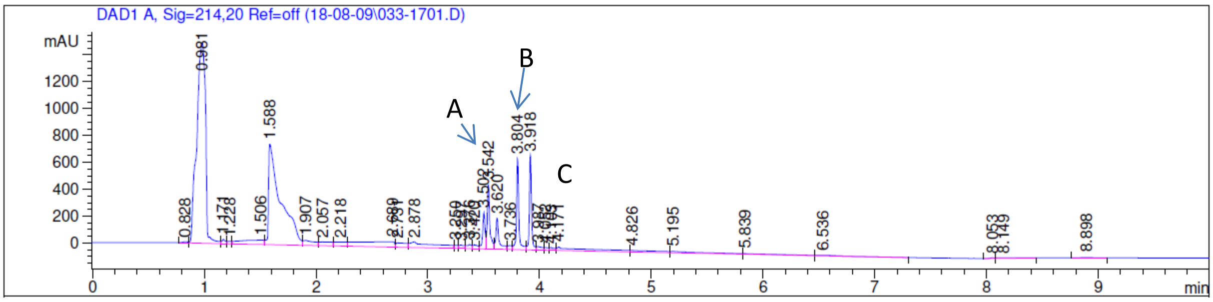

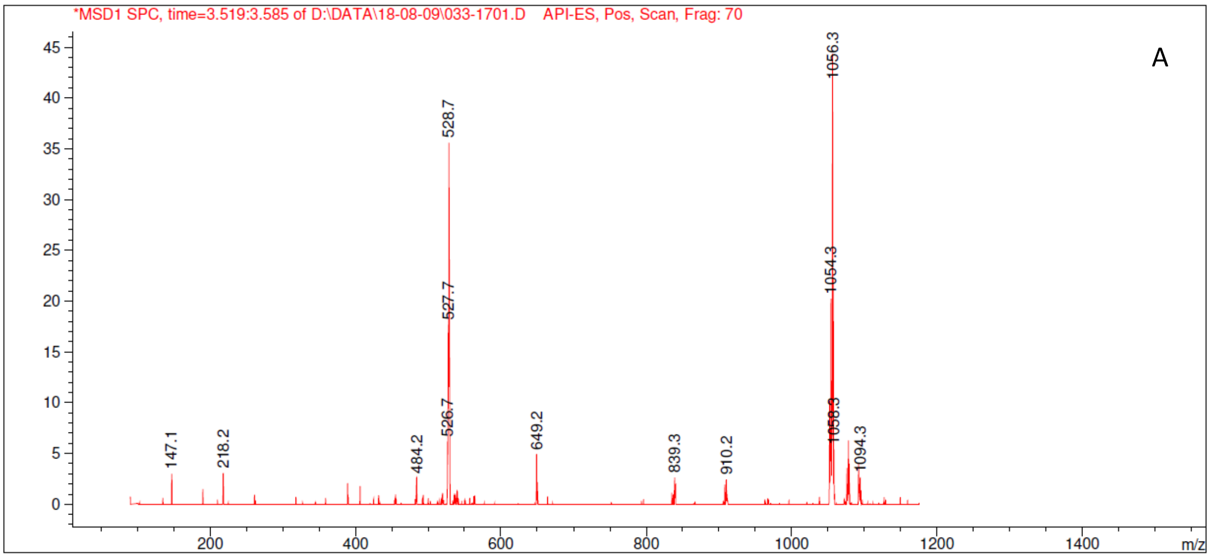

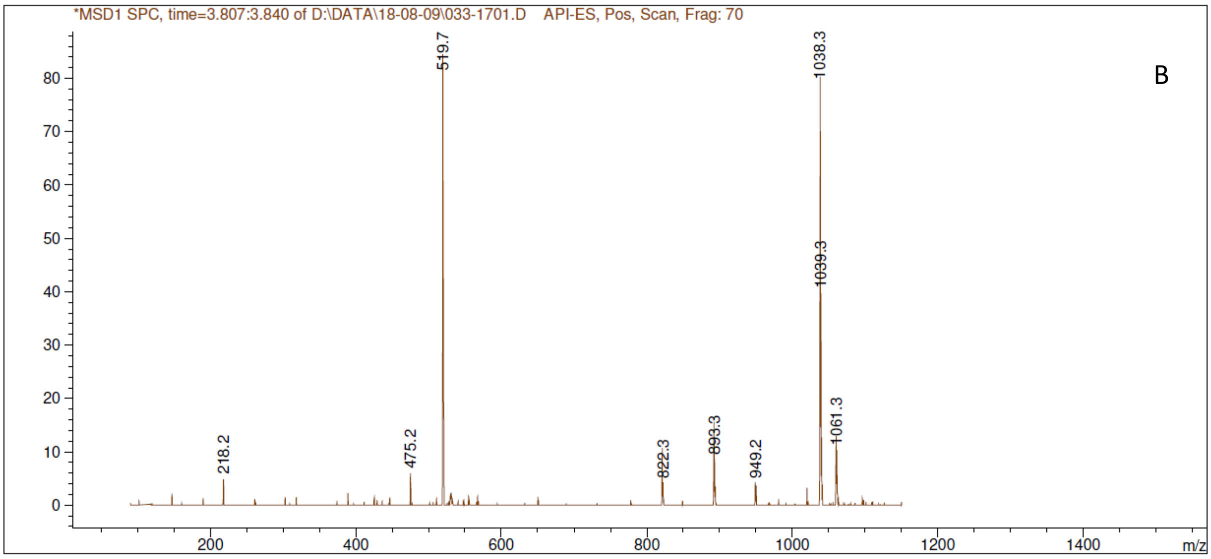

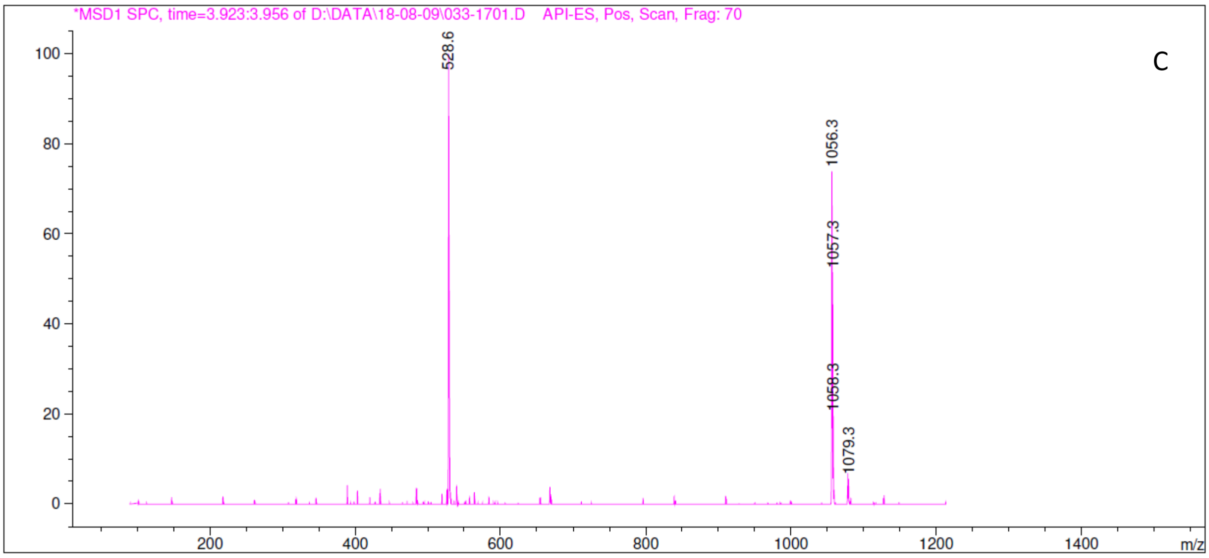

Figure S10.3. LC chromatogram at 214 nm for one pot oxidation / reduction of peptide **5K** (Exact mass = 1055.5) to **7K** and ESI-MS (positive mode) spectra for peaks A, B and C.

Peak A: oxidation + imine formation + reduction + H: 1055.5 + 16 – 18 + 2 + 1 = 1056.5
Peak B: oxidation + imine formation + reduction+ pyrrole formation + H: 1055.5 + 16 – 18 + 2 -18 + 1 = 1038.5
Peak C**:** starting material with exact mass = 1055.5

11. NMR analysis of peptide 6K

To confirm the proposed pyrrole linkage for the cyclisation, NMR analysis was performed. Larger scale reaction was carried out and purified via Semi-PrepHPLC Luna C18 column, 0-100 ACN in 15 min.

The 1D ^{1}H and 2D ^{1}H-^{1}H, ^{1}H-^{13}C experiments were recorded on a Bruker 700.13MHz Avance II spectrometer equipped with a 5 mm ^{1}H/^{19}F ^{13}C ^{15}N Prodigy TCI cryoprobe. For each sample measurement, the sample temperature was set at 25° (and controlled within ± 0.1 °c with a Eurotherm 2000 VT controller. The spectra recorded on the samples included 1D ^{1}H, ^{1}H-{^{1}H} TOCSY (80ms spinlock), ^{1}H-{^{13}C} HSQC and ^{1}H-{^{13}C} HMBC spectra with an 8Hz long range coupling constant and ^{1}H-{^{1}H} off-resonance ROESY (200ms spinlock). The 2D 1H spectra were recorded with excitation sculpting as the method of choice for the suppression of the residual ^{1}H H_2O signal. No ^{13}C spectra were recorded. All spectra were processed using TOPSPIN 3.5 pl7. All spectra were referenced with respect to the internal TSP standard signal, residing at 0ppm. Standard pulse programs from the Bruker pulse program library were used throughout. The dry sample (±1mg) were dissolved in ± 600 µl 90%/10% H_2O/D_2O (99,96% D) solvent mixture + 0,05mM TSP (Trimethylsilylpropanoic acid) for internal chemical shift referencing.

Assignment of the gathered NMR data (Table S 11.1) comfirmed the proposed pyrrole structure. ROESY signals indicate that the proton on C5 of the pyrrole unit is in close proximity (<5 Å) to two CH_2 protons of the lysine side chain. On the other hand TOCSY cross peaks of low intensity were identified between the CH_2 of the former furylalanine and the proton on the C3 of the pyrrole unit. These findings confirm the covalent connection of the pyrrole unit to the peptide backbone on positions 4 and 10.

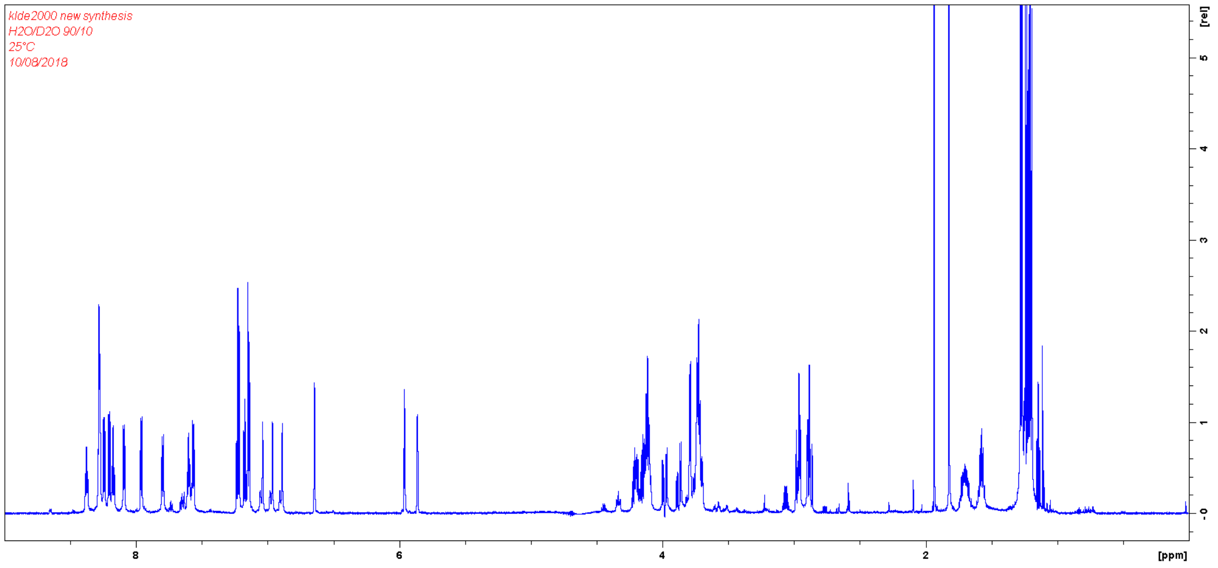

Figure S11.1. ^{1}H NMR spectrum of peptide **6K** dissolved in 90/10 H_2O/D_2O.

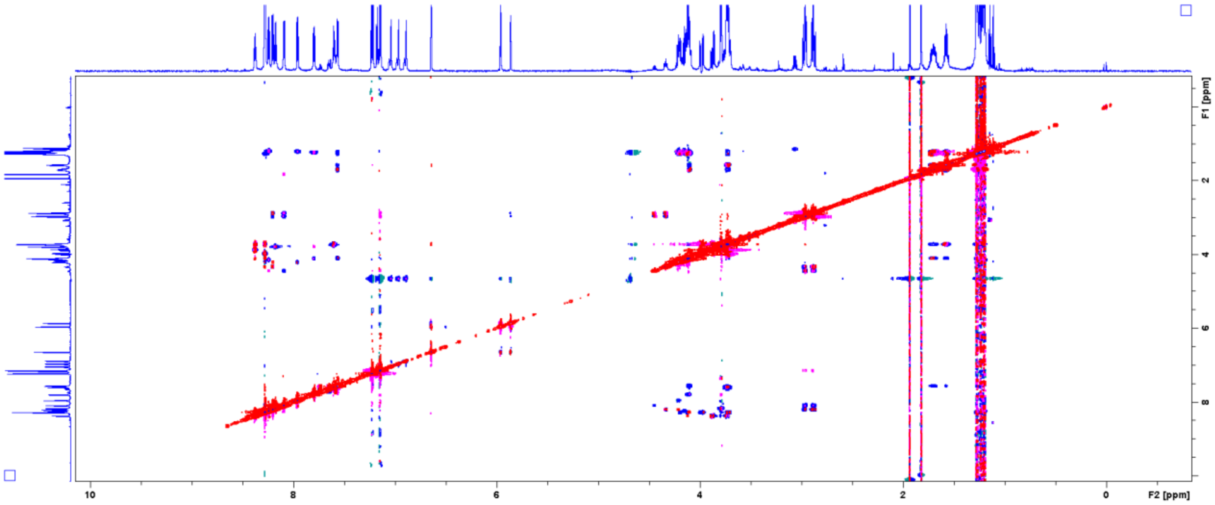

Figure S11.2. TOCSY (blue/green) and ROESY (red/pink) overlay 2D NMR spectrum of peptide **6K** dissolved in 90/10 H_2O/D_2O.

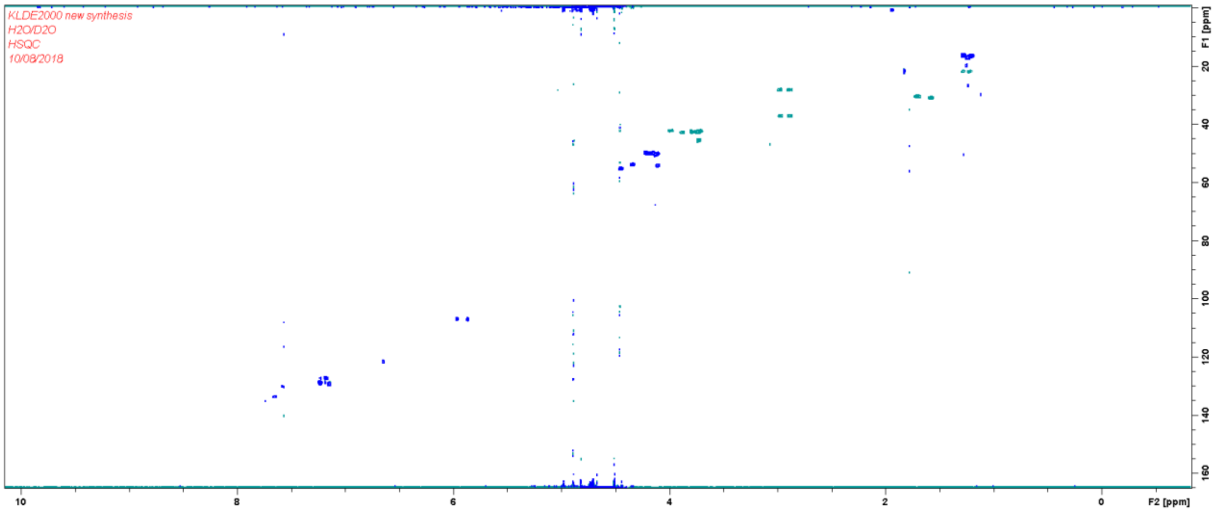

Figure S11.3. HSQC 2D NMR spectrum of peptide **6K** dissolved in 90/10 H_2O/D_2O .

Table S11.1. ^{1}H and ^{13}C assignment of peptide **6K** (dissolved in 90/10 H_2O/D_2O, 700 MHz). Residues 4 and 10, where the pyrrole unit is connected to the peptide backbone, are marked with an asterisk. Carbon atoms in the pyrrole unit and proline are numbered as in figure S 11.4.

residue		^{1}H δ (ppm)	^{13}C δ (ppm)	residue		^{1}H δ (ppm)	^{13}C δ (ppm)
Phe 1	Cα	4.3311	53.7453	Pro 7	$_5CH_2$	3.0691	46.7821
	CH_2	2.9273	36.9783				
	NH	8.2					
				Gly 8	Cα	3.7929	42.3807
Ala 2	Cα	4.1196	50.3152		NH	8.17	
	CH_3	1.2759	16.2085				
	NH	8.28		Ala 9	Cα	4.1090	50.1739
					CH_3	1.2381	17.0170
Gly 3	Cα	3.9873/3.7146	42.09		NH	7.8	
	NH	8.28					
				Lys * 10	Cα	4.1019	54.1592
Pyrrole * 4	Cα	4.4465	55.1382		$C\beta H_2$	1.2205/1.2798	21.7659
	CH_2	2.9766/2.8801	27.9836		$C\gamma/\delta H_2$	1.5753	30.8094
	$_5CH$	6.6454	121.5040		$C\gamma/\delta H_2$	1.6998	30.3496
	$_4CH$	5.9622	106.9262		NH	7.56	
	$_3CH$	5.8646	106.9940				
	NH	8.09		Gly 11	Cα	3.7259	45.4140
					NH	7.6	
Ala 5	Cα	4.1528	49.7401				
	CH_3	1.1962	16.3453	Ala 12	Cα	4.2177	49.6695
	NH	8.24			CH_3	1.2101	16.4448
					NH	7.96	
Gly 6	Cα	3.7327/3.8788	42.4232				
	NH	8.37					

Chemical Formula: $C_{49}H_{69}N_{13}O_{14}$
Exact Mass: 1063,51
Molecular Weight: 1064,17

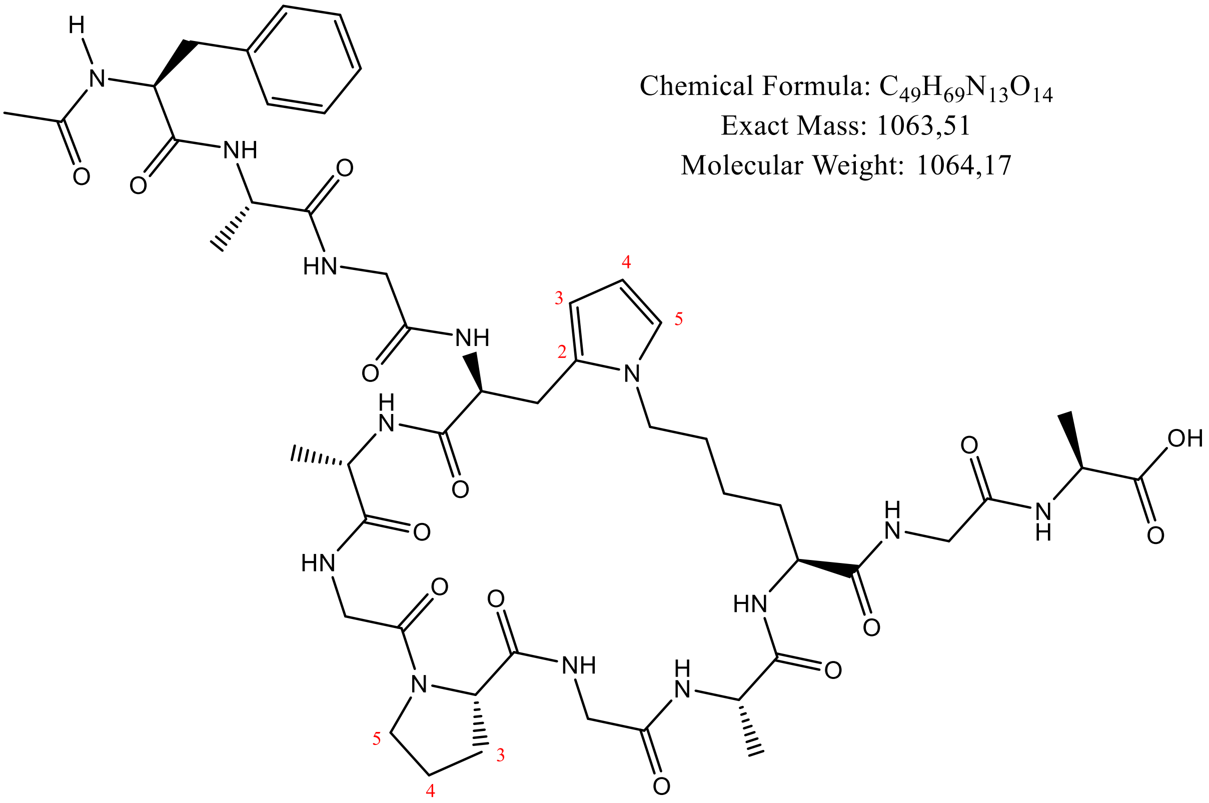

Figure S11.4. Structural representation of peptide **6K**.

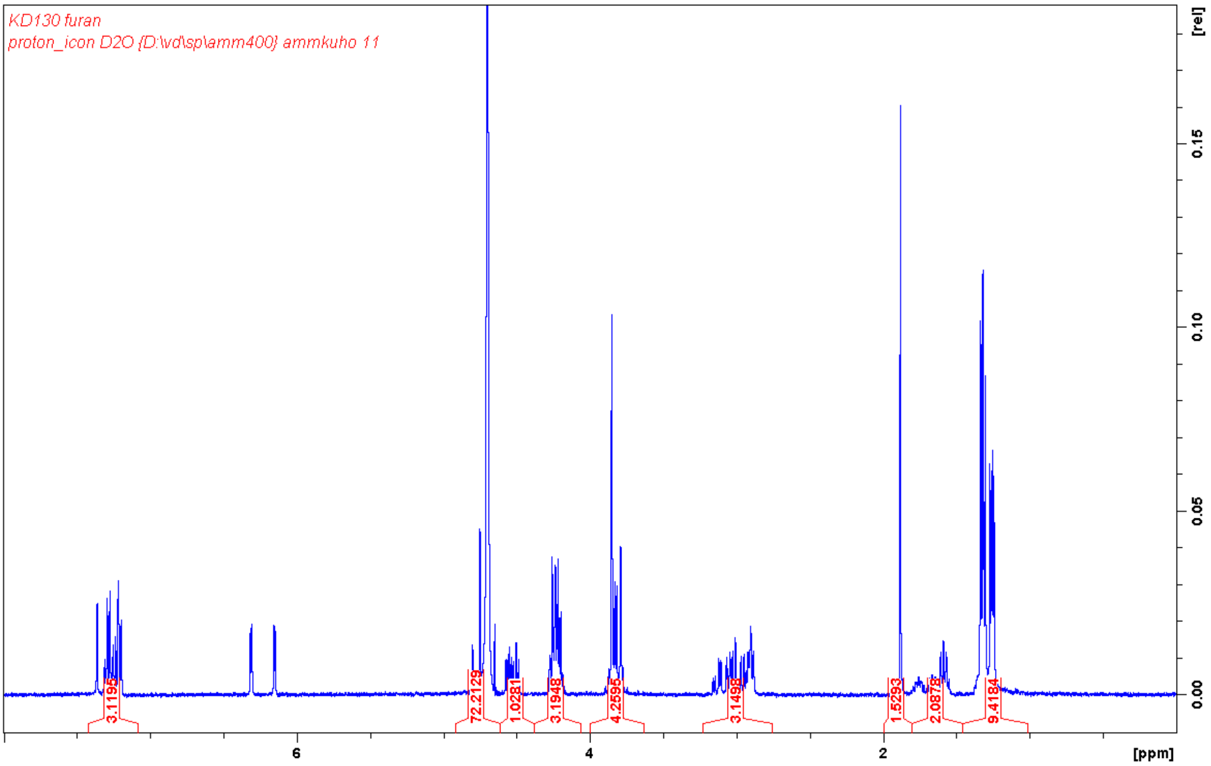

Figure S11.5. ^{1}H NMR spectrum of peptide **4K** dissolved in D_2O. Measured on 400 MHz Bruker equipment.

12. Catalogue numbers of products and instruments

Table S12.1. Catalogue numbers of products

Product	Catalogue number
Chlorotrityl resin	BR-1060 (Iris Biotech)
Boc-L-furylalanine.DCHA	09804 (Sigma-Aldrich)
Fmoc-Gly-OH	FAA1050 (Iris Biotech)
Fmoc-L-Ala-OH	FAA1005 (Iris Biotech)
Fmoc-L-Pro-OH	FAA1185 (Iris Biotech)
Fmoc-L-Phe-OH	FAA1175 (Iris Biotech)
Fmoc-L-Ala(2-furyl)-OH	FAA4250 (Iris Biotech)
Fmoc-L-Lys(boc)-OH	FAA1125 (Iris Biotech)
Fmoc-L-Ser(tbu)-OH	FAA1190 (Iris Biotech)
HBTU	RL-1030 (Iris Biotech)
DIPEA	387649 (Sigma-Aldrich)
Piperidine	104094 (Sigma-Aldrich)
TFA	SOL-011 (Iris Biotech)
chloroacetonitrile	C19651 (Sigma-Aldrich)
Acetic acid anhydride	149490010 (Acros organic)
NaOAc	W302406 (Sigma-Aldrich)
NBS	B81255 (Sigma-Aldrich)
$NaCNBH_3$	156159 (Sigma-Aldrich)

Table S12.2. Catalogue numbers of instruments

instrument	Catalogue number
Bruker UltraFlex MALDI-TOF-MS	-
Pierce C-tip purification tips	87782 (Thermo Fisher)
Intavis MultiPep RSi	-
Multisyntech Syro	-
Agilent 1100 Series HPLC instrument	-
Phenomenex Luna C18 (2) column	00G-4252-E0 (Phenomenex)
Bruker 700.13MHz Avance II	-
